# Supplementary material for: Systemic circulating microRNA landscape in Lynch syndrome
Source: Int J Cancer. 2022 Nov 2;152(5):932–44. doi: 10.1002/ijc.34338 (PMC10092425; doi:10.1002/ijc.34338)
Supplement: Supplementary file 1 — File S1. Supporting Information [file IJC-152-932-s001.pdf]

Supplementary material

Systemic circulating microRNA landscape in Lynch syndrome

Tero Sievänen, Tia-Marje Korhonen, Tiina Jokela, Maarit Ahtiainen, Laura Lahtinen, Teijo Kuopio, Anna Lepistö, Elina Sillanpää, Jukka-Pekka Mecklin, Toni T. Seppälä & Eija K. Laakkonen

Correspondence to: Tero Sievänen, tero.o.sievanen@jyu.fi

Table of content

1. Supplementary materials & methods 2
   1.1. Small-RNA sequencing 2
   1.2. MicroRNA discovery 2
   1.3. RT-qPCR validation 2
   1.4. HCT116 cell line experiment 3
2. Supplementary tables 4
3. Supplementary figures 7
4. Supplementary excel-files and R-code are available as separate files 11
   4.1. Supplementary file 2 11
   4.2. Supplementary file 3 11
   4.3. Data availability 11

1. Supplementary materials & methods

1.1. Small-RNA sequencing

The summary statistics of all sequencing runs are listed in Table S1. Samples that had < 1M raw reads were excluded from the downstream analysis (n=5). A total of 155 samples were sequenced successfully in three separate sequencing runs with minor under-clustering affecting the runs. The mean raw read count of the experiment was 3,761,804 M reads per sample and after adapter removal, trimming and filtering of low-quality data the mean clean read count was 1,601,265 M reads per sample. On average, 1,150,017 clean reads per sample mapped to human microRNAs (miRs) resulting in mean alignment rate of 63 %.

1.2. MicroRNA discovery

The alignment produced 1349 distinct c-miRs within the discovery cohort (supplementary file 2). After filtering out the genes with low expression (<1 count per million in 70% of samples per treatment group), 228 c-miRs were left for differential expression analysis (supplementary file 2). All the 228 c-miRs were observed in all three treatment groups (Figure S1). Top 5 c-miRs with the highest raw counts (highest sequencing depth) as a proportion of all c-miR counts per sample for Lynch syndrome (LS), sporadic rectal cancer patients (SRME) and control (CTRL) groups are presented in Figures S2, S3 and S4, respectively. Hsa-miR-16-5p had the highest raw count in all groups followed by hsa-let-7a-5p, hsa-let-7b-5p, hsa-miR-122-5p (SRME only) and hsa-miR-223-3p and hsa-miR-451a.

1.3. RT-qPCR validation

RT-qPCR validation of the selected differentially expressed c-miRs was performed using independent validation cohort that is independent from discovery and cancer cohorts. Validation cohort (n=29) comprised of 14 healthy *path_MMR* carriers and 15 non-LS controls (Table S3). DE c-miRs identified in the discovery cohort with log2 fold change being significantly above or below the average and with mean count >100 counts were chosen to validate the sequencing results using an independent validation cohort. These inclusion criteria were chosen to increase the probability of detecting c-miRs with RT-qPCR which is less sensitive and more prone to noise than sequencing. After small-RNA isolation, cDNA was synthetized with miRCURY®LNA® RT kit (339340, Qiagen). cDNA synthesis was done from 12 µl of non-diluted template RNA and PCR protocol was carried out in standard thermocycler (Eppendorf). Transcripts levels were measured by using miRCURY LNA™ miRNA PCR assays (has-let-7e-5p, hsa-miR-141-3p, hsa-miR-155-5p, hsa-miR-206, hsa-miR-320a, hsa-miR-339-5p, hsa-miR-451a, hsa-miR-484, hsa-miR-3613-5p, 339350, Qiagen) and miRCURY LNA SYBR® Green kit (339346, Qiagen). One µl of 1:2 diluted cDNA was used per well and samples were run as triplicates. qPCR protocol was the following: 95°C (2 min, activation), 95°C (10 s), 56°C (60 s) with 40 cycles (CFX384™ Real-Time PCR Detection System, Bio-Rad). Fold expression was calculated using the formula 2(-ΔΔCt), where ΔCt(sample)- ΔCt (mean Ct from all samples), ΔCt is Ct (c-miR of interest)-Ct (mean Ct from the group) and Ct is the cycle at which the detection threshold is crossed. Samples with Ct values >35 were excluded from the analysis.

Gene expression of the selected c-miRs (hsa-let-7e-5p, hsa-miR-141-3p, hsa-miR-155-5p, hsa-miR-206, hsa-miR-320a, hsa-miR-339-5p, hsa-miR-451a, hsa-miR-484 and hsa-miR-3613-5p) did not display statistically significant differences between the discovery and validation cohorts (Fig. S5A). Pearson correlations of the c-miR expression fold changes between the discovery and validation cohorts showed that seven out of nine RT-qPCR validated c-miRs had corresponding fold changes in both cohorts ($p<0.001$, $r = 0.985$). Two c-miRs, hsa-miR-206 and -3613-5p, showed deviations (Fig. S5B). Hsa-miR-206 had positive fold change in both cohorts but the magnitude of the fold change value was substantially higher in the validation cohort compared to the discovery cohort. The fold change of hsa-miR-3613-5p differentiated between the cohorts by showing downregulation in discovery cohort but upregulation in validation cohort (Fig. S5B). Taken together, c-miR expression fold changes followed an analogous trend in both cohorts in 8 out of 9 validation c-miRs. The substantial variation in the overall c-miR expression within the validation cohort due to small sample size could have affected the statistical power and thus failed to verify our findings.

1.4. HCT116 cell line experiment

The c-miR-target gene interactions we reported in our manuscript are already confirmed with experimental evidence (MiRTarBase). However, as we cannot completely exclude the possibility that these interactions differ among LS and sporadic cancer, we performed a small-scale cell line experiment using parental human colorectal cell line (HCT116) (RRID:CVCL_0291, purchased from Horizon Discovery Ltd., UK, 30.3.2016) which possess microsatellite instability to mimic LS colorectal cancer. HCT116 cell line has not been authenticated using STR profiling by the authors within the last three years. All experiments were conducted with mycoplasma-free cells. We examined whether overexpression of hsa-miR-122b-3p (as mimic) or underexpression of hsa-miR-451a (as inhibitor) have an impact on HCT116 cells' viability or apoptosis. These miRs were selected as representatives of over- and underexpressed miRs found in healthy LS carriers vs non-LS control comparisons. HCT116 was cultured in RPMI medium (Sigma), supplemented with 10% fetal bovine serum (Gibco), 2mM GlutaMAX (Gibco) and 50 units/ml penicillin and 50mg/ml streptomycin (Gibco). Cells were plated on 96-well plate for cell viability and apoptosis assays. After 24h, cells were transfected using Lipofectamine™ 3000 Transfection Reagent (Cat#L3000008, Thermo Fisher), and negative control-miR (Cat#4464058, Thermo Fisher), miR 122b-3p-mimic (Cat#MC21073, Thermo Fisher) or miR-451a-inhibitor (Cat#MH10286, Thermo Fisher). After 48h incubation cell viability was analyzed with Cell titer Glo 2.0 (Cat# G9242, Promega) whereas apoptosis was analyzed by using RealTime-Glo Annexin V apoptosis and Necrosis assay (Cat#JA1011, Promega) according to the manufacturer's instructions. We performed three independent transfection experiments to overexpress miR-122b-3p and underexpress miR-451a in HCT116 cells. Each experiment included 4-6 technical replicants. Cell viability and apoptosis was measured from the mean luminescence of three experiments. Kruskal-Wallis test was used for statistical testing. We did not find significant differences ($p > 0.05$) in mean luminescence when comparing Opti-MEM (no treatment) and c-miR-122b-3p overexpression or hsa-miR-451a underexpression. Nevertheless, a trend towards reduced cell viability due to apoptosis appear credible (Figure S6A-C).

2. Supplementary tables

This section contains all the supplementary tables.

Table S1. Sequencing summary statistics of all sequencing runs.

SEQUENCING RUN I

Parameter	Lynch syndrome	Sporadic rectal cancer patient	Control	Total
N	41	10	10	61
Raw read count (mean[count], ± SD)	2,997,064 (677,455)	3,341,588 (1,586,332)	3,508,312 (1,180,659)	3,282,321 (455,310)
Clean read count (mean[count], ± SD)	1,760,483 (518,578)	1,123,486 (542,040)	1,814,271 (820,051)	1,566,080 (167,693)
MicroRNA-aligned read count (mean[count], ± SD)	1,009,995 (447,449)	678,927 (324,164)	1,079,379 (592,904)	922,767 (134,522)
Alignment-% (mean [%])	55.5 %	61.0 %	57.5 %	58.0 %

SEQUENCING RUN II

Parameter	Lynch syndrome	Sporadic rectal cancer patient	Control	Total
N	24	6	7	37
Raw read count (mean[count], ± SD)	3,850,746 (1,514,056)	4,593,992 (743,092)	3,796,429 (1,298,281)	4,080,389 (397,739)
Clean read count (mean[count], ± SD)	1,444,305 (950,828)	1,090,272 (624,565)	1,732,170 (924,363)	1,422,249 (181,212)
MicroRNA-aligned read count (mean[count], ± SD)	983,377 (686,158)	669,817 (401,775)	1,113,962 (688,652)	922,385 (164,913)
Alignment-% (mean [%])	65.8 %	58.9 %	63.2 %	62.6 %

SEQUENCING RUN III

Parameter	Lynch syndrome	Sporadic rectal cancer patient	Control	Total
N	29	8	20	57
Raw read count (mean[count], ± SD)	3,884,176 (1,663,080)	4,605,843 (1,008,139)	4,782,816 (1,419,636)	4,424,278 (331,044)
Clean read count (mean[count], ± SD)	1,675,657 (1,190,841)	1,256,684 (554,348)	1,733,051 (1,134,394)	1,555,131 (352,317)
MicroRNA-aligned read count (mean[count], ± SD)	1,219,297 (1,005,301)	708,023 (388,872)	1,256,710 (862,318)	1,061,344 (322,640)
Alignment-% (mean [%])	70.5%	57.0%	71.2%	66.2 %

SEQUENCING RUN I-III				
**Parameter**	**Lynch syndrome**	**Sporadic rectal cancer patient**	**Control**	**Total**
N	94	24	37	155
Raw read count (mean[count], ± SD)	3,577,329 (531,282)	4,180,474 (431,202)	4,029,185 (119,493)	3,761,804 (1,395,080)
Clean read count (mean[count], ± SD)	1,626,815 (340,682)	1,156,814 (44,520)	1,759,831 (160,107)	1,601,265 (886,932)
MicroRNA-aligned read count (mean[count], ± SD)	1,070,890 (279,891)	685,589 (41,587)	1,150,017 (136,572)	1,035,926 (698,458)
Alignment-% (mean [%])	63.9 %	59.0 %	64.0 %	63.0 %

Table S2. Non-differentially expressed c-miRs within and between the discovery and cancer cohorts.

**Sporadic rectal cancer patients vs *path_MMR with cancer***			***Path_MMR* with cancer vs non-LS control**		
miR	log2FC	FDR	miR	log2FC	FDR
hsa-let-7a-5p	0.009	0.984	hsa-mir-10b-5p	0.688	0.268
hsa-let-7b-5p	0.065	0.984	hsa-mir-125a-5p	0.603	0.268
hsa-let-7c-5p	0.223	0.984	hsa-mir-127-3p	-1.643	0.268
hsa-let-7d-3p	0.295	0.984	hsa-mir-144-3p	-0.959	0.268
hsa-let-7d-5p	-0.229	0.984	hsa-mir-16-5p	0.863	0.268
hsa-let-7e-5p	0.261	0.984	hsa-mir-32-5p	-0.749	0.268
hsa-let-7f-5p	0.237	0.984	hsa-mir-361-3p	0.913	0.268
hsa-let-7g-5p	0.195	0.984	hsa-mir-423-3p	-0.603	0.268
hsa-let-7i-5p	-0.109	0.984	hsa-mir-15b-5p	-0.760	0.271
hsa-mir-100-5p	-0.585	0.984	hsa-mir-374a-5p	-1.014	0.271
hsa-mir-101-3p	-0.255	0.984	hsa-mir-181d-5p	-1.257	0.280
hsa-mir-103a-3p	0.136	0.984	hsa-mir-15a-5p	-0.736	0.322
hsa-mir-103b	0.107	0.984	hsa-mir-190a-5p	-0.695	0.322
hsa-mir-106b-3p	-0.593	0.984	hsa-mir-320a-3p	-0.649	0.322
hsa-mir-106b-5p	0.460	0.984	hsa-mir-370-3p	-1.287	0.322
hsa-mir-107	0.262	0.984	hsa-mir-432-5p	-0.930	0.322
hsa-mir-10a-5p	0.284	0.984	hsa-mir-451a	-0.730	0.322
hsa-mir-10b-5p	0.143	0.984	hsa-mir-654-3p	-1.401	0.322
hsa-mir-11400	-0.085	0.984	hsa-mir-107	-0.615	0.339
hsa-mir-1180-3p	0.076	0.984	hsa-mir-221-3p	-0.389	0.339

Table S3. Descriptive characteristics of study subjects in the validation cohort.

Variable		Validation cohort	
		***Path_MMR*, healthy**	**non-LS, healthy**
**N**		14	15
**Sex** (N (%))			
	Male	6 (42.9)	8 (53.3)
	Female	8 (57.1)	7 (46.7)
**Age,** years (mean± SD)		59.7 (11.9)	57.3 (15.1)
**Body mass index,** kg/m^2 (mean± SD)$^{\#}$		28.6 (7.9)	30.7 (5.1)
Path_MMR (N (%))			
	MLH1	14 (100.0)	-
	MSH2	0 (0.0)	-
	MSH6	0 (0.0)	-
	PMS2	0 (0.0)	-
**Previous cancers** (N (%))			
	Yes	9 (64.2)	-
	No	5 (35.8)	-

3. Supplementary figures

This section contains all the supplementary figures.

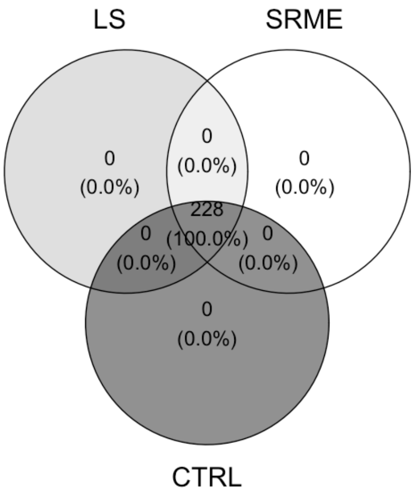

Figure S1. Venn diagram of c-miR distribution among treatment groups. LS = Lynch syndrome, SRME = sporadic rectal cancer patients, CTRL = control.

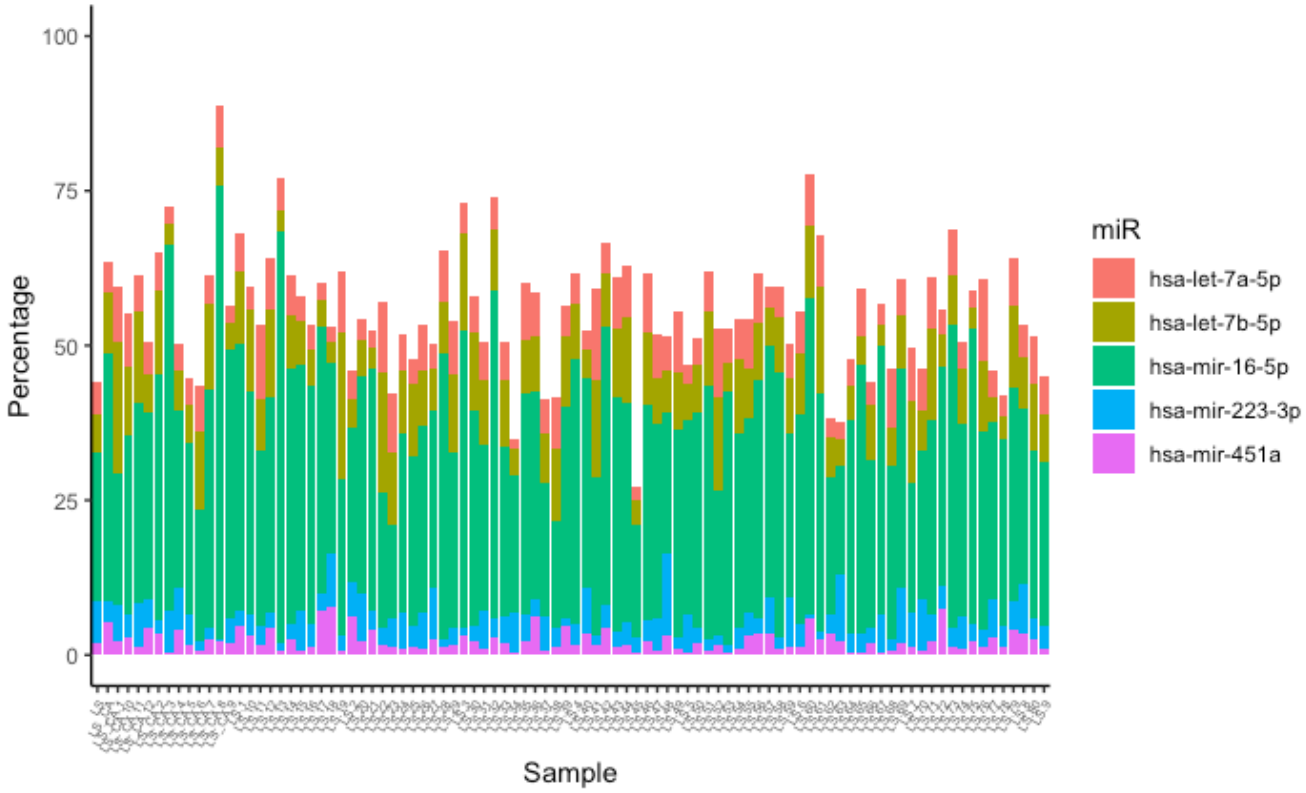

Figure S2. Top 5 c-miRs with the highest raw counts (highest sequencing depth) as a proportion of all c-miR counts per sample in Lynch syndrome group. Samples are shown on the x-axis and percentages on the y-axis.

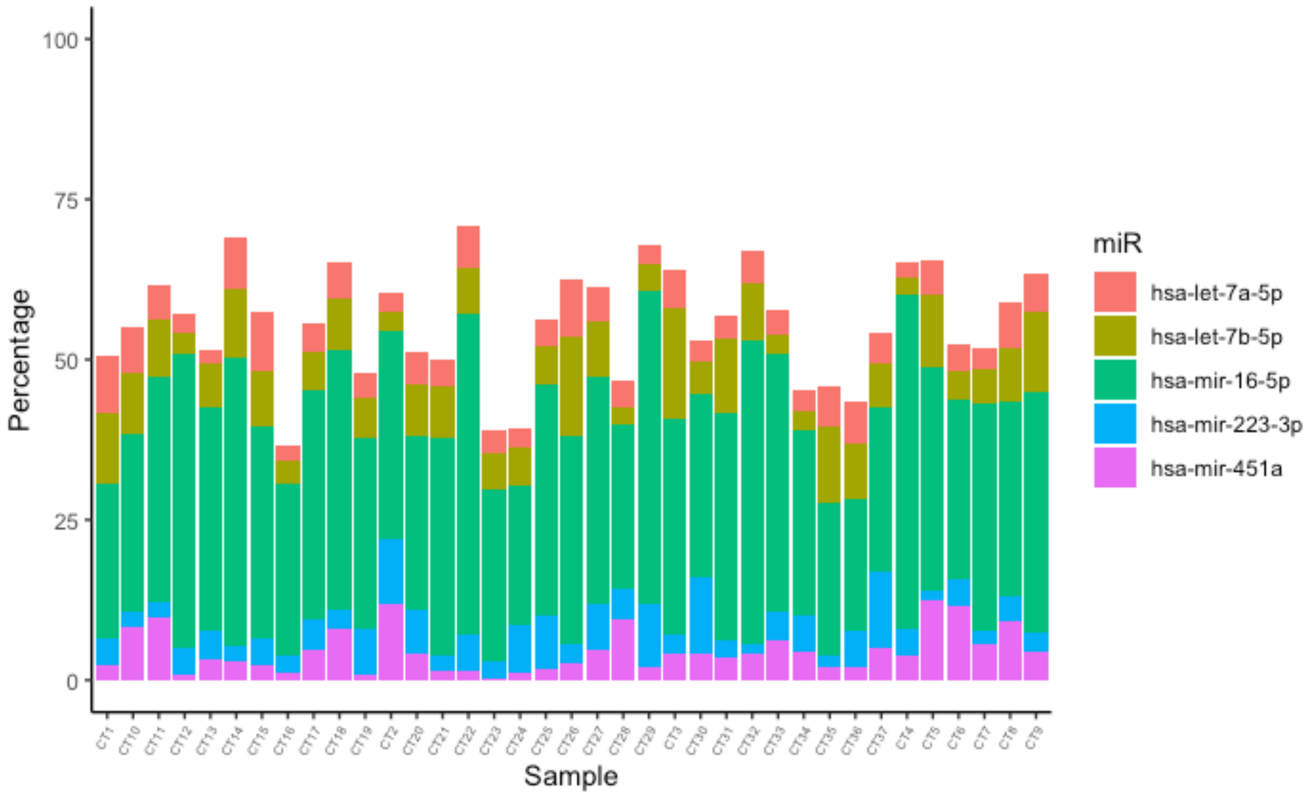

Figure S3. Top 5 c-miRs with the highest raw counts (highest sequencing depth) as a proportion of all c-miR counts per sample in control group. Samples are shown on the x-axis and percentages on the y-axis.

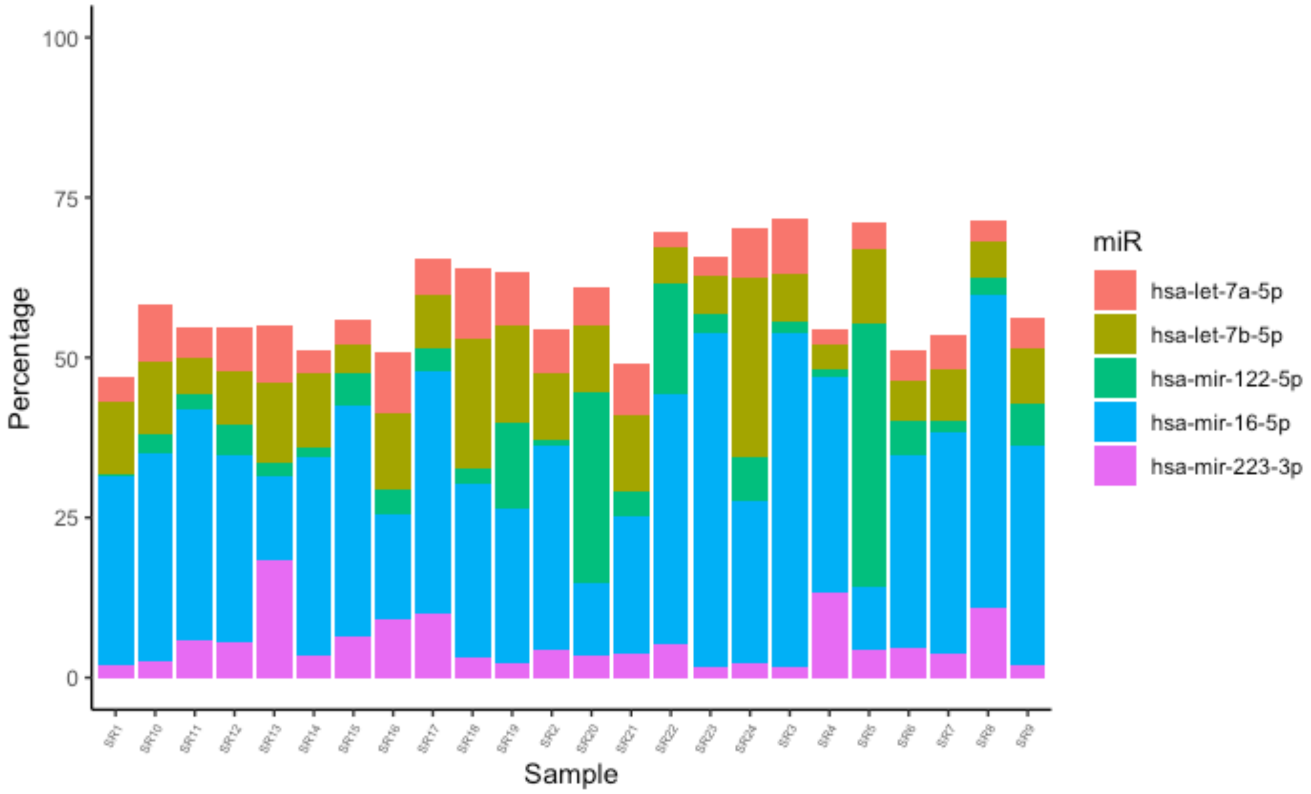

Figure S4. Top 5 c-miRs with the highest raw counts (highest sequencing depth) as a proportion of all c-miR counts per sample in sporadic rectal cancer patient group. Samples are shown on the x-axis and percentages on the y-axis.

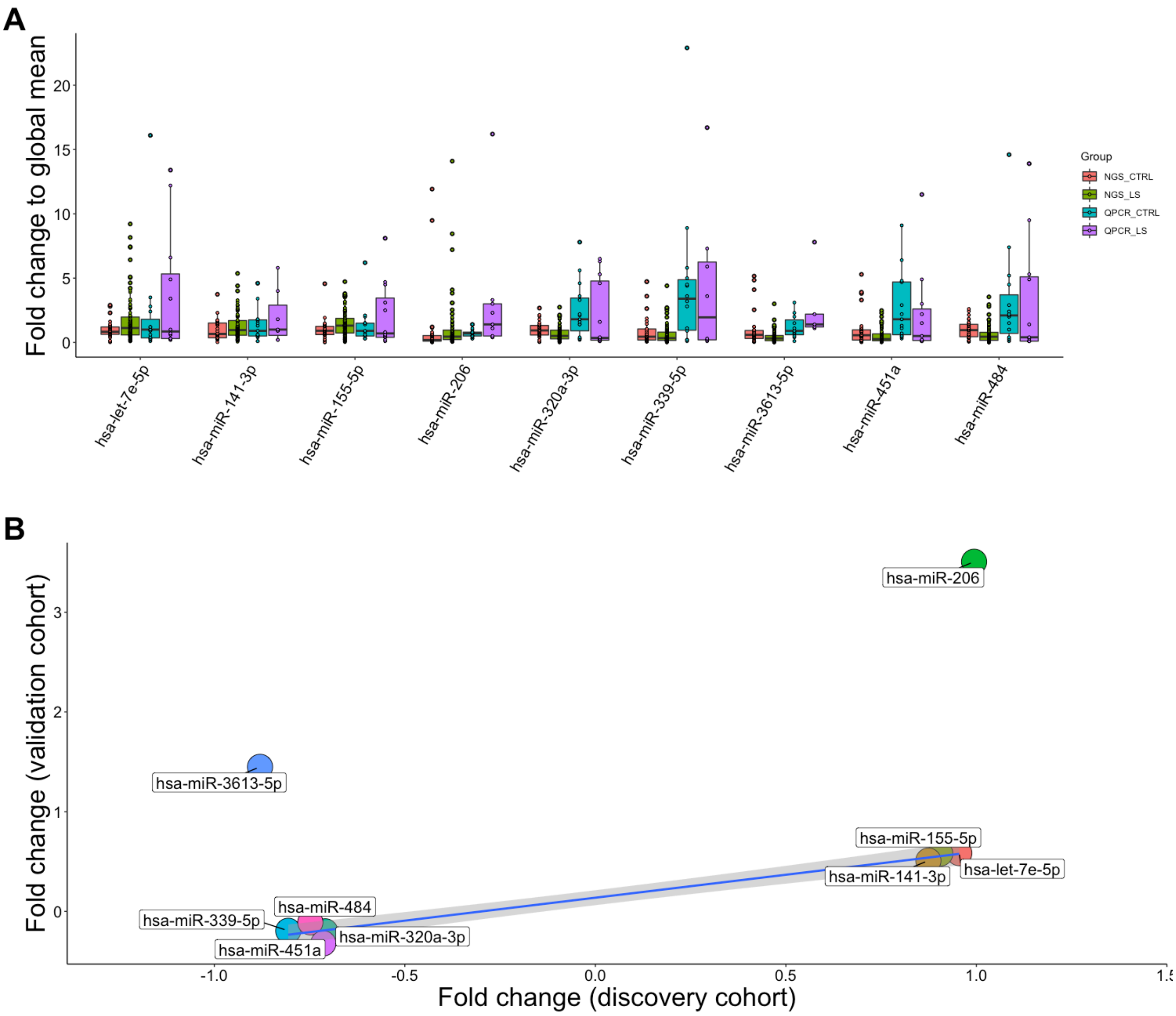

Figure S5. RT-qPCR validation revealed significant variation in c-miR expression in the validation cohort. **A,** distributions of expression fold changes of the nine selected c-miRs compared to global mean expression between the discovery and validation cohorts. Error bars represent SEM. Red = non-LS control samples from discovery cohort; Green = healthy *path_MMR* carriers from discovery cohort; Cyan = non-LS control samples from validation cohort; Purple = *path_MMR* carriers from validation cohort. **B,** Pearson correlations of expression fold changes of selected c-miRs between the discovery and validation cohorts. Blue line indicates the linear model fit of the 7 c-miRs which correlated between the experiments and grey cloud represents variation. c-miR = circulating microRNA.

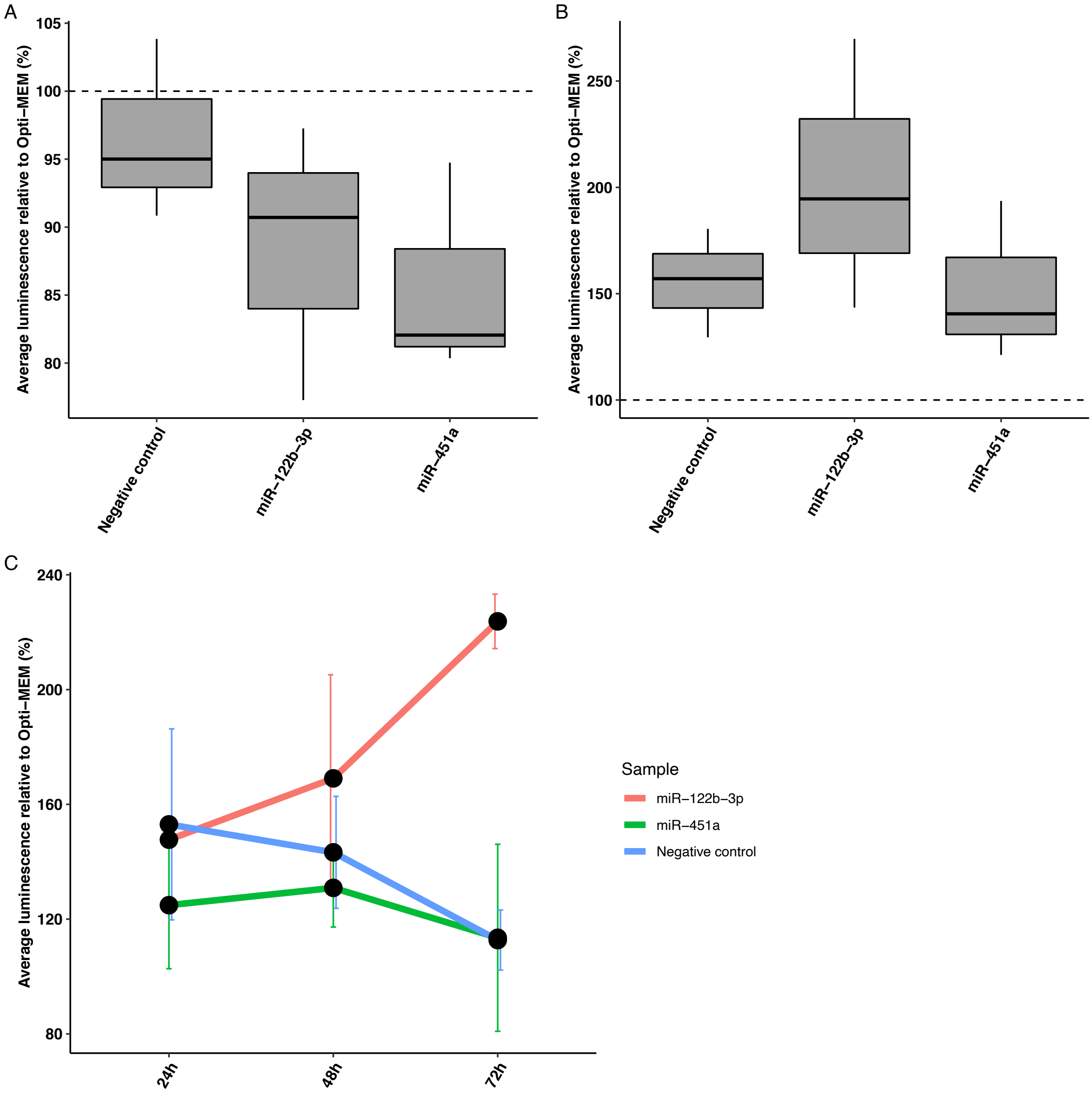

Figure S6. Cell viability and apoptosis assay results with HCT116 cell line. **A)** The impact of overexpression of hsa-miR-122b and underexpression of hsa-miR-451a on HCT116 cell viability (n=3) presented as average luminescence (%) compared to Opti-MEM. **B)** The impact of overexpression of hsa-miR-122b and underexpression of hsa-miR-451a on HCT116 cell apoptosis (n=3). presented as average luminescence (%) compared to Opti-MEM. **C)** Apoptosis due to overexpression of hsa-miR-122b and underexpression hsa-miR-451a on HCT116 cells (n=3) at three distinct timepoints: 24h, 48h and 72h, presented as average luminescence compared to Opti-MEM with standard deviation. Dashed line = Opti-MEM. Opti-MEM = no treatment. Negative control = transfection with scrambled miR.

4. Supplementary excel-files and R-code are available as separate files

4.1. Supplementary file 2

Supplementary excel file include raw and filtered c-miR count data, c-miR-target gene prediction data and pathway analysis (GO and KEGG) data.

4.2. Supplementary file 3

Supplementary code file include all the R-scripts used for differential expression testing, all the used R-packages and their dependencies as well as the operating system information.

4.3. Data availability

The datasets are available in the Gene Expression Omnibus (GSE198834) and from the corresponding author upon request. Step-by-step analysis protocols can be accessed via GitHub repository (https://zenodo.org/badge/latestdoi/467491700).
