# Supplementary material for: Systemic circulating microRNA landscape in Lynch syndrome
Source: Int J Cancer. 2022 Nov 2;152(5):932–44. doi: 10.1002/ijc.34338 (PMC10092425; doi:10.1002/ijc.34338)
Supplement: Supplementary file 3 — File S3. Supporting Information [file IJC-152-932-s002.pdf]

Code supplementary file

Tero Sievänen & Tia-Marje Korhonen

2022-04-26

Contents

Introduction 1

R-packages used 1

Contact information 2

Preprocessing and read mapping 2

Preprocessing . . . 2

Read mapping . . . 2

Analysis 2

Step 1 - C-miR count data filtering . . . 2

Step 2 - Sex difference within *path_MMR* carriers, sporadic rectal cancer patients, and non-LS control group . . . 3

Step 3 - *Path_MMR* variants in LS . . . 6

Step 4 - Cancer history in *path_MMR* carriers . . . 8

Step 5 - Healthy *path_MMR* carriers vs *path_MMR* carriers with cancer . . . 10

Step 6 - Healthy *path_MMR* carriers vs non-LS control group . . . 12

Step 7 - Sporadic rectal cancer patients vs healthy *path_MMR* carriers . . . 15

Step 8 - Sporadic rectal cancer patients vs *path_MMR* carriers with cancer . . . 17

Step 9 - Sporadic rectal cancer patients vs non-LS control group . . . 19

Step 10 - *Path_MMR* carriers with cancer vs non-LS control group . . . 22

Session info 24

Introduction

This code supplementary file was used to perform all differential expression analyses between and within the discovery and cancer cohorts.

R-packages used

A variety of R packages was used for this analysis. All packages used are available from the Comprehensive R Archive Network (CRAN), Bioconductor.org, or Github etc.

Contact information

All the scripts have been written by Tero Sievänen (Uni. of Jyväskylä) and Tia-Marje Korhonen (Uni. of Jyväskylä).

Correspondence to: tero.o.sievanen@jyu.fi

Preprocessing and read mapping

Preprocessing

FastQC was used for sequence quality control throughout the pipeline. Sequencing adapter removal, trimming and filtering was done with FASTX-Toolkit.

Read mapping

Bowtie aligner was used for mapping the preprocessed high-quality reads to human miR-genome derived from miRBase v.22. A cut-off of >=1M reads per sample was applied before bioinformatic analysis. Mean raw read count was 3,761,804 M and 1,082,304 M was the mean c-miR read count across all samples. The mean alignment rate throughout the study population was 63%.

Analysis

Step 1 - C-miR count data filtering

This script is used to make a filtered c-miR raw count file. Genes with low counts must be removed before DE-analysis, since their biological significance is low or these genes might be false positives.

```r
# Load packages
library(edgeR) # Package for differential gene expression analysis of RNA-seq data

# Import raw counts file
counts <- read.csv("rawCounts.tsv",header=TRUE,sep="\t")

# Import study design file
targets<- read.csv("phenodata.txt",sep="\t",header=TRUE)

# Add column names
colnames(counts) <-targets$Filename

# Create a digital gene expression list (DGEList) using edgeR
myDGEList <- DGEList(counts = counts)

# Convert DGEList to counts per million (cpm)
cpm <- cpm(myDGEList)

# Keep only the c-miRs with >1 CPM in at least 70% of samples in a subgroup
keepers <- rowSums(cpm>1)>=108  #70% of 155

# Create a new filtered DGEList
myDGEList.filtered <- myDGEList[(keepers),]
```

```
# Create a new filtered raw counts matrix
FilteredCounts <- counts[which(rownames(counts)%in%rownames(myDGEList.filtered$counts)),]

# Create a new .txt file of filtered raw miR counts
write.table(FilteredCounts,"FilteredCounts.txt",sep="\t")
```

Step 2 - Sex difference within *path_MMR* carriers, sporadic rectal cancer patients, and non-LS control group

This script is used to perform DE-analysis between sexes among *path_MMR* carriers, sporadic rectal cancer patients (SRME) and non-LS control group (CTRL).

```
# Load packages
library(tidyverse) # Tidyverse is an opinionated collection of R packages designed for data science
library(gt) # Package for making static data tables
library(DESeq2) # Package for differential gene expression analysis of RNA-seq data

# Read in the filtered c-miR counts table from step 1
counts <- read.csv("FilteredCounts.txt",header=TRUE,sep="\t")

# Add sample names (column names) to raw counts table
colnames(counts) <-targets$Filename

# Choose only the healthy path_MMR carriers (!= cancer | future_ca), n = 81
select <- which(targets$Type=="LS" & targets$Healthy_now=="YES")

# AND/ OR
#select <- which(targets$Type=="SRME") # No sex difference
#select <- which(targets$Type=="CTRL") # No sex difference

# Create a new filtered counts file
Counts <- counts[,select]

# New phenofile with only the variables of interest
Targets <- targets[select,]

# Setup design matrix for DE-analysis
condition <- as.character(Targets$Sex) # Condition of interest
batch <- as.character(Targets$NGS) # Batch effect
group_levels <- levels(as.factor(condition))
design <- data.frame(condition=as.factor(condition), batch=batch) # DE-design for the analysis
rownames(design) <- colnames(Counts)
dds <- DESeqDataSetFromMatrix(countData=Counts, colData=design, design = ~ batch + condition)

# DESeq2 DE-analysis of the condition of interest, batch effect taken into account
dds <- DESeq(dds)

# Display results
res <- results(dds, alpha=0.05) # Statistical significance at the level p=< 0.05
resOrdered <- res[order(res$padj),] # Order results based on adjusted p-value
summary(res)
```

```
##
```

```
## out of 228 with nonzero total read count
## adjusted p-value < 0.05
## LFC > 0 (up)       : 0, 0%
## LFC < 0 (down)     : 2, 0.88%
## outliers [1]       : 0, 0%
## low counts [2]     : 0, 0%
## (mean count < 8)
## [1] see 'cooksCutoff' argument of ?results
## [2] see 'independentFiltering' argument of ?results
```

```
resOrdered
```

```
## log2 fold change (MLE): condition m vs f
## Wald test p-value: condition m vs f
## DataFrame with 228 rows and 6 columns
##                         baseMean      log2FoldChange             lfcSE
##                        <numeric>           <numeric>         <numeric>
## hsa-mir-206     307.713556384502    -1.33068564558869  0.31505843163435
## hsa-mir-223-5p   459.11883569172   -0.615199198839776 0.169101214163155
## hsa-mir-25-3p   4820.42085197208    0.430107971473196 0.135801218563743
## hsa-mir-10a-5p  346.157958154898   -0.461120240691358 0.156566195466845
## hsa-mir-200a-3p 80.6044955883249    -1.09287077881835 0.370468286286468
## ...                          ...                  ...               ...
## hsa-mir-660-5p  155.677865957598   0.0147634968852977 0.257280472213827
## hsa-mir-92b-3p  64.5712400305805  -0.0213800813149473 0.314517885127516
## hsa-mir-339-5p  107.204731203206  0.00414465840788723 0.266443362717535
## hsa-mir-141-3p  185.044728072799  -0.0010733625846962 0.338906004582831
## hsa-mir-664a-5p 95.2512944422679 -0.00139353456409118 0.282058993696403
##                                stat               pvalue                padj
##                           <numeric>            <numeric>           <numeric>
## hsa-mir-206        -4.22361540583385 2.40414173602933e-05 0.00548144315814688
## hsa-mir-223-5p     -3.63805311442773 0.000274706780777468  0.0313165730086313
## hsa-mir-25-3p       3.16718786489615  0.00153920848027874   0.116979844501184
## hsa-mir-10a-5p     -2.94520946438279  0.00322736091714395   0.117641431481693
## hsa-mir-200a-3p    -2.94997121014908  0.00317803541903545   0.117641431481693
## ...                              ...                  ...                 ...
## hsa-mir-660-5p    0.0573828894134945    0.954240192811382   0.989272592642855
## hsa-mir-92b-3p   -0.0679773148871299    0.945803692602105   0.989272592642855
## hsa-mir-339-5p    0.0155554950426035     0.98758901119346   0.996328736956234
## hsa-mir-141-3p  -0.00316713947283828    0.997472992537353   0.997472992537353
## hsa-mir-664a-5p -0.00494057837273263    0.996058004831867   0.997472992537353
```

```
mcols(res)$description
```

```
## [1] "mean of normalized counts for all samples"
## [2] "log2 fold change (MLE): condition m vs f"
## [3] "standard error: condition m vs f"
## [4] "Wald statistic: condition m vs f"
## [5] "Wald test p-value: condition m vs f"
## [6] "BH adjusted p-values"
```

```
# Create a data frame of the ordered results (top 20)
padj.subset <- head(resOrdered, 20) %>%
  as_tibble(rownames = "miR")

# Create a gene table of the subset
```

```r
gt(padj.subset)
```

miR	baseMean	log2FoldChange	lfcSE	stat	pvalue	padj
hsa-mir-206	307.71356	-1.3306856	0.3150584	-4.223615	2.404142e-05	0.005481443
hsa-mir-223-5p	459.11884	-0.6151992	0.1691012	-3.638053	2.747068e-04	0.031316573
hsa-mir-25-3p	4820.42085	0.4301080	0.1358012	3.167188	1.539208e-03	0.116979845
hsa-mir-10a-5p	346.15796	-0.4611202	0.1565662	-2.945209	3.227361e-03	0.117641431
hsa-mir-200a-3p	80.60450	-1.0928708	0.3704683	-2.949971	3.178035e-03	0.117641431
hsa-mir-221-3p	1598.95610	-0.3810347	0.1309301	-2.910215	3.611798e-03	0.117641431
hsa-mir-223-3p	32698.88794	-0.4419576	0.1507263	-2.932187	3.365840e-03	0.117641431
hsa-mir-101-3p	1120.60950	0.5711740	0.2167622	2.635025	8.413100e-03	0.191818675
hsa-mir-16-5p	303422.44373	0.4261250	0.1598380	2.665980	7.676424e-03	0.191818675
hsa-mir-375-3p	133.75566	-0.8895514	0.3333510	-2.668513	7.618788e-03	0.191818675
hsa-mir-3135b	118.94662	-0.7310454	0.2826903	-2.586029	9.708873e-03	0.201238450
hsa-let-7c-5p	1064.66630	-0.3893307	0.1587407	-2.452620	1.418201e-02	0.231098489
hsa-mir-106b-5p	65.25566	0.9493544	0.3815972	2.487844	1.285200e-02	0.231098489
hsa-mir-122-5p	56331.29965	-0.7427463	0.3083287	-2.408943	1.599878e-02	0.231098489
hsa-mir-19b-3p	533.22070	0.3801106	0.1552042	2.449100	1.432136e-02	0.231098489
hsa-mir-574-5p	70.34374	-0.8648140	0.3597418	-2.403985	1.621744e-02	0.231098489
hsa-mir-215-5p	36.58455	-1.1671429	0.4936453	-2.364335	1.806247e-02	0.242249592
hsa-mir-28-3p	195.99300	-0.4363951	0.1902585	-2.293696	2.180799e-02	0.262504821
hsa-mir-99a-5p	101.91519	-0.6783997	0.2959182	-2.292524	2.187540e-02	0.262504821
hsa-mir-10b-5p	394.00082	-0.3102299	0.1405471	-2.207302	2.729299e-02	0.311140067

```r
# Create a data frame of all results for plotting
res.df <- as_tibble(res, rownames = "miR")

# Create a volcano plot of results
ggplot(data=res.df,
  aes(y=-log10(res$padj), x=res$log2FoldChange, text = paste(rownames(Counts)))) +
  xlab("Log2FC") +
ylab("-log10(Padj)") +
  geom_point(size=2) +
  geom_hline(yintercept = -log10(0.05), linetype="longdash", colour="grey", size=1) +
  geom_vline(xintercept = 1, linetype="longdash", colour="#BE684D", size=1) +
  geom_vline(xintercept = -1, linetype="longdash", colour="#2C467A", size=1) +
  labs(title="Volcano plot",
       subtitle = "Men vs women in LS group",
       caption=paste0("produced on ", Sys.time())) +
  theme_bw()
```

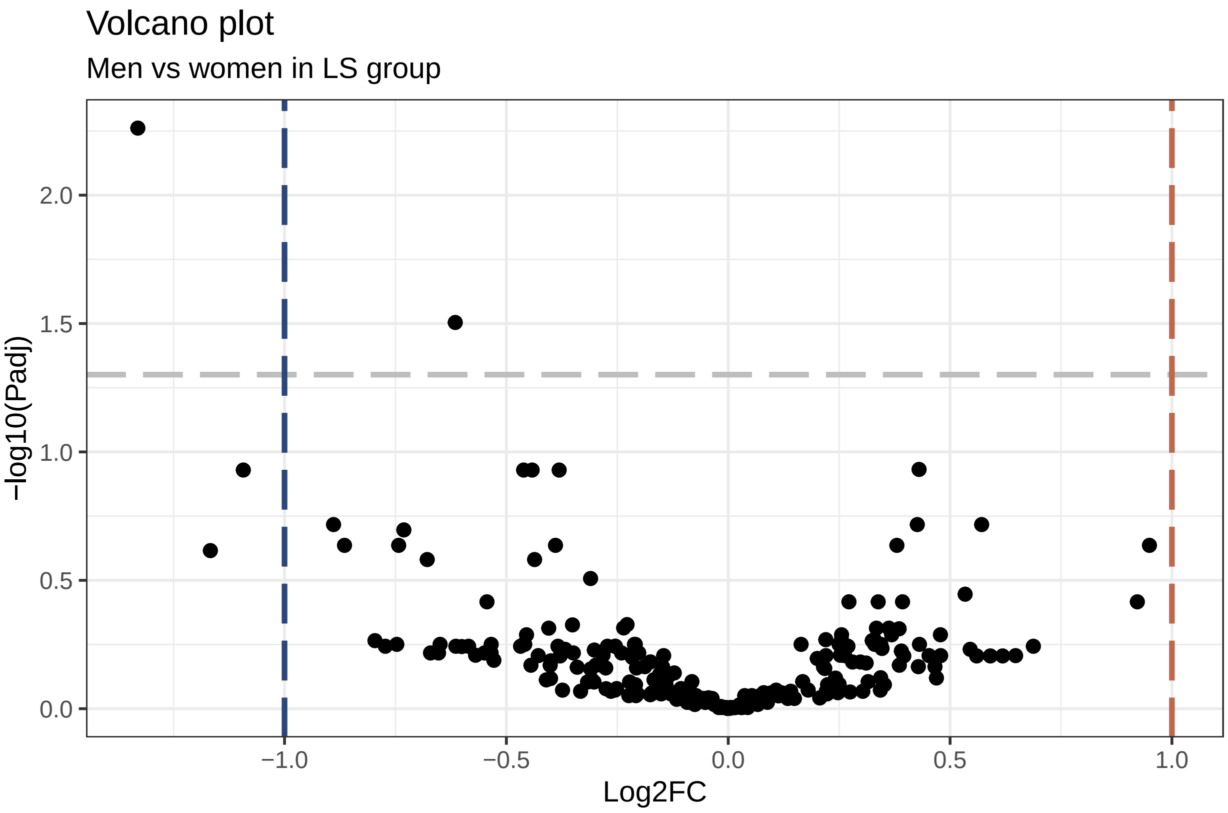


```
# We saw that there is a sex difference within LS group, so to confirm that this is an unique finding, 

# Run the script again from the beginning but subset the groups from phenodata
```

Step 3 - *Path_MMR* variants in LS

This script is used to perform DE-analysis between MLH1 and other variants (MSH2, MSH6 and PMS2) among *path_MMR* carriers.

```
# Setup design matrix for DE-analysis
condition <- as.character(Targets$step_3) # condition of interest (path_MMR variant)
batch <- as.character (Targets$NGS) # Batch effect
sex <- as.character(Targets$Sex) # Sex as covariate
group_levels <- levels(as.factor(condition))
design <- data.frame(condition=as.factor(condition), batch=batch, sex=sex)
rownames(design) <- colnames(Counts)
dds <- DESeqDataSetFromMatrix(countData=Counts, colData=design, design = ~ batch + sex + condition)

# DESeq2 DE-analysis of the condition of interest, batch effect and sex as covariates
dds <- DESeq(dds)

# Display results
res <- results(dds, alpha=0.05)
resOrdered <- res[order(res$padj),]
summary(res)
```

```
##
## out of 228 with nonzero total read count
## adjusted p-value < 0.05
## LFC > 0 (up)       : 0, 0%
## LFC < 0 (down)     : 0, 0%
## outliers [1]       : 0, 0%
## low counts [2]     : 0, 0%
## (mean count < 8)
## [1] see 'cooksCutoff' argument of ?results
## [2] see 'independentFiltering' argument of ?results
```

```
resOrdered
```

```
## log2 fold change (MLE): condition YES vs NO
## Wald test p-value: condition YES vs NO
## DataFrame with 228 rows and 6 columns
##                          baseMean       log2FoldChange             lfcSE
##                         <numeric>            <numeric>         <numeric>
## hsa-mir-206      307.713556384502    -0.918700279522276 0.317688589739701
## hsa-mir-382-5p   480.873186468193     -0.89617112870515 0.300633964174415
## hsa-mir-3065-5p   28.929340476312     0.832735664944019 0.305878893197249
## hsa-mir-155-5p   719.638264871698     0.487418932393798 0.188214918704313
## hsa-mir-185-5p   1172.42293127207    -0.327633576073769 0.132678647051713
## ...                           ...                  ...               ...
## hsa-mir-3613-5p 148.694191498994    0.0126353013852315 0.288019149661537
## hsa-mir-423-5p   7564.1850783196   0.00586543133990573 0.197717936854708
## hsa-mir-664a-5p 95.2512944422679    0.0163241981203909 0.294919727552127
## hsa-mir-20a-5p  872.103417280602 -0.000792319956047674 0.191890092408413
## hsa-mir-361-5p  333.398661677954 -0.000487762461682228 0.131883549541673
##                                stat              pvalue              padj
##                           <numeric>           <numeric>         <numeric>
## hsa-mir-206       -2.89182649044781 0.00383009405770217 0.436630722578048
## hsa-mir-382-5p    -2.98093773657999 0.00287367213209918 0.436630722578048
## hsa-mir-3065-5p    2.72243585112955 0.00648026131387386 0.492499859854413
## hsa-mir-155-5p     2.58969339810696 0.00960614468691482 0.547550247154145
## hsa-mir-185-5p    -2.46937682403461  0.0135348606646302 0.617189646307139
## ...                             ...                 ...               ...
## hsa-mir-3613-5p   0.043869657278274    0.96500830199003 0.996259041239742
## hsa-mir-423-5p   0.0296656511453279   0.976333706272898 0.996259041239742
## hsa-mir-664a-5p  0.0553513264639296   0.955858572203358 0.996259041239742
## hsa-mir-20a-5p  -0.00412903004059909  0.996705520040721 0.997049084198545
## hsa-mir-361-5p  -0.00369843292341856  0.997049084198545 0.997049084198545
```

```
mcols(res)$description
```

```
## [1] "mean of normalized counts for all samples"
## [2] "log2 fold change (MLE): condition YES vs NO"
## [3] "standard error: condition YES vs NO"
## [4] "Wald statistic: condition YES vs NO"
## [5] "Wald test p-value: condition YES vs NO"
## [6] "BH adjusted p-values"
```

```
# Create a data frame of the ordered results (top 20)
subset <- head(resOrdered, 20) %>%
  as_tibble(rownames = "miR")
```

```r
# Create a gene table of the subset
gt(subset)
```

miR	baseMean	log2FoldChange	lfcSE	stat	pvalue	padj
hsa-mir-206	307.71356	-0.91870028	0.3176886	-2.8918265	0.003830094	0.4366307
hsa-mir-382-5p	480.87319	-0.89617113	0.3006340	-2.9809377	0.002873672	0.4366307
hsa-mir-3065-5p	28.92934	0.83273566	0.3058789	2.7224359	0.006480261	0.4924999
hsa-mir-155-5p	719.63826	0.48741893	0.1882149	2.5896934	0.009606145	0.5475502
hsa-mir-185-5p	1172.42293	-0.32763358	0.1326786	-2.4693768	0.013534861	0.6171896
hsa-mir-22-3p	785.73921	-0.36713202	0.1549583	-2.3692306	0.017825136	0.6773552
hsa-let-7a-5p	55093.52537	0.07330551	0.1767418	0.4147604	0.678317285	0.9730676
hsa-let-7c-5p	1064.66630	-0.19312682	0.1647984	-1.1718973	0.241238279	0.9730676
hsa-let-7d-3p	205.22656	-0.19022601	0.2483986	-0.7658096	0.443789578	0.9730676
hsa-let-7f-5p	27096.20520	0.12854418	0.1304796	0.9851669	0.324542076	0.9730676
hsa-let-7g-5p	3442.14965	-0.09148421	0.1776490	-0.5149718	0.606572717	0.9730676
hsa-let-7i-5p	24698.63988	0.09293477	0.1315752	0.7063245	0.479986354	0.9730676
hsa-mir-101-3p	1120.60950	0.21284126	0.2255534	0.9436401	0.345353602	0.9730676
hsa-mir-103a-3p	2938.41602	0.05112773	0.1278369	0.3999450	0.689197034	0.9730676
hsa-mir-103b	1023.43521	0.04833881	0.1296346	0.3728850	0.709234031	0.9730676
hsa-mir-106b-3p	206.50278	-0.28818442	0.2205021	-1.3069465	0.191230871	0.9730676
hsa-mir-106b-5p	65.25566	-0.36266101	0.3971767	-0.9130975	0.361191277	0.9730676
hsa-mir-107	166.46817	-0.25047445	0.1729971	-1.4478538	0.147657941	0.9730676
hsa-mir-10b-5p	394.00082	-0.03916555	0.1469321	-0.2665555	0.789811447	0.9730676
hsa-mir-11400	46.33394	-0.14462079	0.4377049	-0.3304070	0.741092419	0.9730676

Step 4 - Cancer history in *path_MMR* carriers

This script is used to perform DE-analysis between *path_MMR carriers* with or without previous cancer(s).

```r
# Setup design matrix for DE-analysis
condition <- as.character (Targets$Previous_ca) # Condition of interest (cancer history, 0 = no, 1 = ye
batch <- as.character (Targets$NGS) # Batch effect
sex <- as.character(Targets$Sex) # Sex as covariate
group_levels <- levels(as.factor(condition))
design <- data.frame(condition=as.factor(condition), batch=batch, sex=sex)
rownames(design) <- colnames(Counts)
dds <- DESeqDataSetFromMatrix(countData=Counts, colData=design, design = ~ batch + sex + condition)

# DESeq2 DE-analysis of the condition of interest, batch effect and sex as covariates
dds <- DESeq(dds)

# Display results
res <- results(dds, alpha=0.05)
resOrdered <- res[order(res$padj),]
summary(res)
```

```
##
## out of 228 with nonzero total read count
## adjusted p-value < 0.05
## LFC > 0 (up)       : 0, 0%
## LFC < 0 (down)     : 0, 0%
## outliers [1]       : 0, 0%
```

```
## low counts [2]     : 0, 0%
## (mean count < 8)
## [1] see 'cooksCutoff' argument of ?results
## [2] see 'independentFiltering' argument of ?results
```

```
resOrdered
```

```
## log2 fold change (MLE): condition 1 vs 0
## Wald test p-value: condition 1 vs 0
## DataFrame with 228 rows and 6 columns
##                        baseMean      log2FoldChange             lfcSE
##                       <numeric>           <numeric>         <numeric>
## hsa-mir-206    307.713556384502    0.923234747428217   0.3042997072089
## hsa-mir-140-3p 153.441332464748     0.69732033975536 0.261657008482432
## hsa-mir-224-5p 380.133634872442    0.777869288843377  0.32485293193053
## hsa-mir-375-3p  133.75566338306   -0.790275906805975  0.32494477377035
## hsa-let-7a-5p  55093.5253734347    0.187963807864038 0.168589326071645
## ...                         ...                 ...               ...
## hsa-mir-671-5p 20.9477534559676   0.0198160870856095 0.451436218964841
## hsa-mir-93-5p  4722.36954674415 -0.00739775157996786 0.135343368357135
## hsa-mir-942-5p 47.6943912997957  -0.0763723738706613 0.416039815635458
## hsa-mir-95-3p  31.9363719789938   0.0551554343302459 0.393108998040146
## hsa-mir-30e-3p 165.140350431392  0.00183674883319789 0.263439159317336
##                               stat             pvalue              padj
##                          <numeric>          <numeric>         <numeric>
## hsa-mir-206       3.03396528342507 0.00241362203166191 0.550305823218916
## hsa-mir-140-3p    2.66501686234091  0.0076984462488131 0.877622872364694
## hsa-mir-224-5p    2.39452753041406  0.0166417940627821 0.948582261578581
## hsa-mir-375-3p   -2.43203144225514  0.0150144038690078 0.948582261578581
## hsa-let-7a-5p     1.11492116519975   0.264884205972972 0.969981647919622
## ...                            ...                 ...               ...
## hsa-mir-671-5p  0.0438956518177662    0.96498758130869 0.992728816443445
## hsa-mir-93-5p  -0.0546591360165293   0.956410025400524 0.992728816443445
## hsa-mir-942-5p   -0.18356986759551   0.854350903544122 0.992728816443445
## hsa-mir-95-3p    0.140305703011696   0.888418458546679 0.992728816443445
## hsa-mir-30e-3p 0.00697219364789029   0.994437039404086 0.994437039404086
```

```
mcols(res)$description
```

```
## [1] "mean of normalized counts for all samples"
## [2] "log2 fold change (MLE): condition 1 vs 0"
## [3] "standard error: condition 1 vs 0"
## [4] "Wald statistic: condition 1 vs 0"
## [5] "Wald test p-value: condition 1 vs 0"
## [6] "BH adjusted p-values"
```

```
# Create a data frame of the ordered results (top 20)
subset <- head(resOrdered, 20) %>%
  as_tibble(rownames = "miR")

# Create a gene table of the subset
gt(subset)
```

miR	baseMean	log2FoldChange	lfcSE	stat	pvalue	padj
hsa-mir-206	307.71356	0.92323475	0.3042997	3.0339653	0.002413622	0.5503058
hsa-mir-140-3p	153.44133	0.69732034	0.2616570	2.6650169	0.007698446	0.8776229

hsa-mir-224-5p	380.13363	0.77786929	0.3248529	2.3945275	0.016641794	0.9485823
hsa-mir-375-3p	133.75566	-0.79027591	0.3249448	-2.4320314	0.015014404	0.9485823
hsa-let-7a-5p	55093.52537	0.18796381	0.1685893	1.1149212	0.264884206	0.9699816
hsa-let-7b-5p	78617.74407	0.19400024	0.1523492	1.2733918	0.202879028	0.9699816
hsa-let-7c-5p	1064.66630	0.25875637	0.1569028	1.6491511	0.099116681	0.9699816
hsa-let-7d-3p	205.22656	0.12760995	0.2388988	0.5341590	0.593231498	0.9699816
hsa-let-7d-5p	1239.02592	0.05446379	0.1453732	0.3746481	0.707922232	0.9699816
hsa-let-7e-5p	965.58129	0.44854080	0.2233915	2.0078685	0.044657268	0.9699816
hsa-let-7f-5p	27096.20520	0.05777348	0.1257485	0.4594366	0.645920641	0.9699816
hsa-let-7g-5p	3442.14965	-0.06325748	0.1705595	-0.3708822	0.710725274	0.9699816
hsa-let-7i-5p	24698.63988	0.04418673	0.1265056	0.3492866	0.726874140	0.9699816
hsa-mir-100-5p	46.51049	-0.27834185	0.4416407	-0.6302450	0.528534280	0.9699816
hsa-mir-101-3p	1120.60950	-0.07714536	0.2172718	-0.3550639	0.722541748	0.9699816
hsa-mir-103a-3p	2938.41602	-0.05941792	0.1225922	-0.4846795	0.627903703	0.9699816
hsa-mir-103b	1023.43521	-0.07940509	0.1241771	-0.6394506	0.522529845	0.9699816
hsa-mir-106b-3p	206.50278	-0.06491278	0.2136906	-0.3037700	0.761303126	0.9699816
hsa-mir-106b-5p	65.25566	-0.15376889	0.3825078	-0.4020020	0.687682575	0.9699816
hsa-mir-10a-5p	346.15796	0.06618768	0.1569210	0.4217899	0.673178350	0.9699816

Step 5 - Healthy *path_MMR* carriers vs *path_MMR* carriers with cancer

This script is used to perform DE-analysis between healthy *path_MMR* carriers (n=81) and *path_MMR* carriers with cancer (n=13).

```
# Choose conditions of interest (healthy path_MMR carriers, n = 81 and path_MMR carriers with cancer, n
select <- which(targets$Type=="LS" & targets$Healthy_now=="YES" | targets$Healthy_now=="NO")

# Create a new filtered counts file
Counts <- counts[,select]

# New phenofile with only the variables of interest
Targets <- targets[select,]

# Setup design matrix for DE-analysis
condition <- as.character (Targets$Healthy_now) # Condition of interest
batch <- as.character (Targets$NGS) # Batch effect
sex <- as.character(Targets$Sex) # Sex as covariate
group_levels <- levels(as.factor(condition))
design <- data.frame(condition=as.factor(condition), batch=batch, sex=sex)
rownames(design) <- colnames(Counts)
dds <- DESeqDataSetFromMatrix(countData=Counts, colData=design, design = ~ batch + sex + condition)

# DESeq2 DE-analysis of the condition of interest, batch effect and sex as covariates
dds <- DESeq(dds)

# Display results
res <- results(dds, alpha=0.05)
resOrdered <- res[order(res$padj),]
summary(res)
```

```
##
## out of 228 with nonzero total read count
## adjusted p-value < 0.05
```

```
## LFC > 0 (up)       : 0, 0%
## LFC < 0 (down)     : 0, 0%
## outliers [1]       : 0, 0%
## low counts [2]     : 0, 0%
## (mean count < 8)
## [1] see 'cooksCutoff' argument of ?results
## [2] see 'independentFiltering' argument of ?results
```

```
resOrdered
```

```
## log2 fold change (MLE): condition YES vs NO
## Wald test p-value: condition YES vs NO
## DataFrame with 228 rows and 6 columns
##                          baseMean        log2FoldChange             lfcSE
##                         <numeric>             <numeric>         <numeric>
## hsa-mir-127-3p  48.0155927505647      1.54757991082434 0.478860956403904
## hsa-let-7b-5p   82367.6769607343    -0.250486443035102 0.217244307939718
## hsa-let-7c-5p   1046.64340989249     0.190973049839557  0.21370151185407
## hsa-let-7d-3p   209.630058542978    -0.145838717995448 0.309838889110817
## hsa-let-7d-5p   1343.69887604826    -0.324719189652028 0.221464701451058
## ...                          ...                   ...               ...
## hsa-mir-484     448.505322877159   0.00216435004940099 0.396810802499483
## hsa-mir-503-5p  32.0124906562595     -0.01357393058526 0.505835299507598
## hsa-mir-652-3p  47.3776761888421 -0.000604709085427502  0.48879803619999
## hsa-mir-664a-5p 96.3727540555979 -0.000729931278471826 0.364405578438736
## hsa-mir-99a-5p  101.327988540091   0.00775182190537004 0.420844743177602
##                                 stat              pvalue              padj
##                            <numeric>           <numeric>         <numeric>
## hsa-mir-127-3p       3.2317938853194 0.00123015753681806 0.280475918394519
## hsa-let-7b-5p      -1.15301728920146   0.248903288182279 0.998288877191795
## hsa-let-7c-5p      0.893643887601349    0.37151245670584 0.998288877191795
## hsa-let-7d-3p     -0.470692101995265   0.637860625298168 0.998288877191795
## hsa-let-7d-5p      -1.46623451739459   0.142584407997209 0.998288877191795
## ...                              ...                 ...               ...
## hsa-mir-484      0.00545436272341352   0.995648069772354 0.999012909454964
## hsa-mir-503-5p   -0.0268346843300051   0.978591589077029 0.999012909454964
## hsa-mir-652-3p  -0.00123713485047654   0.999012909454964 0.999012909454964
## hsa-mir-664a-5p -0.00200307383218213   0.998401779383912 0.999012909454964
## hsa-mir-99a-5p    0.0184196714608805   0.985304059547971 0.999012909454964
```

```
mcols(res)$description
```

```
## [1] "mean of normalized counts for all samples"
## [2] "log2 fold change (MLE): condition YES vs NO"
## [3] "standard error: condition YES vs NO"
## [4] "Wald statistic: condition YES vs NO"
## [5] "Wald test p-value: condition YES vs NO"
## [6] "BH adjusted p-values"
```

```
# Create a data frame of the ordered results (top 20)
subset <- head(resOrdered, 20) %>%
  as_tibble(rownames = "miR")

# Create a gene table of the subset
gt(subset)
```

miR	baseMean	log2FoldChange	lfcSE	stat	pvalue	padj
hsa-mir-127-3p	48.01559	1.54757991	0.4788610	3.2317939	0.001230158	0.2804759
hsa-let-7b-5p	82367.67696	-0.25048644	0.2172443	-1.1530173	0.248903288	0.9982889
hsa-let-7c-5p	1046.64341	0.19097305	0.2137015	0.8936439	0.371512457	0.9982889
hsa-let-7d-3p	209.63006	-0.14583872	0.3098389	-0.4706921	0.637860625	0.9982889
hsa-let-7d-5p	1343.69888	-0.32471919	0.2214647	-1.4662345	0.142584408	0.9982889
hsa-let-7e-5p	926.64673	0.33109997	0.2942949	1.1250620	0.260562767	0.9982889
hsa-let-7f-5p	26808.59858	0.17400238	0.1598255	1.0887025	0.276285084	0.9982889
hsa-let-7i-5p	24851.67228	0.06746493	0.1650282	0.4088084	0.682680275	0.9982889
hsa-mir-100-5p	48.67038	-0.45345588	0.5825199	-0.7784384	0.436310596	0.9982889
hsa-mir-101-3p	1147.85281	0.17065168	0.2953940	0.5777086	0.563460858	0.9982889
hsa-mir-103a-3p	2918.11081	0.24630999	0.1736895	1.4181055	0.156159970	0.9982889
hsa-mir-103b	1015.61076	0.23920375	0.1725654	1.3861625	0.165697280	0.9982889
hsa-mir-106b-3p	215.96717	-0.21582880	0.2886214	-0.7477920	0.454585615	0.9982889
hsa-mir-106b-5p	66.96291	0.53460596	0.5182629	1.0315342	0.302290377	0.9982889
hsa-mir-107	168.30275	0.24345940	0.2546063	0.9562190	0.338961590	0.9982889
hsa-mir-10a-5p	347.95892	-0.19387889	0.2094819	-0.9255162	0.354697482	0.9982889
hsa-mir-10b-5p	400.29913	-0.17038572	0.1877073	-0.9077203	0.364026003	0.9982889
hsa-mir-11400	43.71408	0.54947799	0.5573913	0.9858029	0.324229849	0.9982889
hsa-mir-1180-3p	48.65138	-0.86372484	0.5626079	-1.5352164	0.124730683	0.9982889
hsa-mir-122b-3p	12230.59670	0.05827116	0.4349263	0.1339794	0.893418872	0.9982889

Step 6 - Healthy *path_MMR* carriers vs non-LS control group

This script is used to perform DE-analysis between healthy *path_MMR* carriers and CTRL group.

```
# Choose conditions of interest (healthy path_MMR carriers, n = 81 and CTRL group, n = 37)
select <- which(targets$Type=="LS" & targets$Healthy_now=="YES" | targets$Type=="CTRL")

# Create a new filtered counts file
Counts <- counts[,select]

# New phenofile with only the variables of interest
Targets <- targets[select,]

# Setup design matrix for DE-analysis
condition <- as.character (Targets$Type) # Condition of interest
batch <- as.character (Targets$NGS) # Batch effect
sex <- as.character(Targets$Sex) # Sex as covariate
group_levels <- levels(as.factor(condition))
design <- data.frame(condition=as.factor(condition), batch=batch, sex=sex)
rownames(design) <- colnames(Counts)
dds <- DESeqDataSetFromMatrix(countData=Counts, colData=design, design = ~ batch + sex + condition)

# DESeq2 DE-analysis of the condition of interest, batch effect and sex as covariates
dds <- DESeq(dds)

# Display results
res <- results(dds, alpha=0.05)
resOrdered <- res[order(res$padj),]
summary(res)
```

```
##
## out of 228 with nonzero total read count
## adjusted p-value < 0.05
## LFC > 0 (up)       : 15, 6.6%
## LFC < 0 (down)     : 25, 11%
## outliers [1]       : 0, 0%
## low counts [2]     : 75, 33%
## (mean count < 69)
## [1] see 'cooksCutoff' argument of ?results
## [2] see 'independentFiltering' argument of ?results
```

```
resOrdered
```

```
## log2 fold change (MLE): condition LS vs CTRL
## Wald test p-value: condition LS vs CTRL
## DataFrame with 228 rows and 6 columns
##                          baseMean     log2FoldChange             lfcSE
##                         <numeric>          <numeric>         <numeric>
## hsa-mir-155-5p  649.461599394047  0.905013618668636 0.169010672683695
## hsa-let-7c-5p   978.123000672281  0.728728381283735 0.145105235191548
## hsa-let-7e-5p   859.891695461067  0.955495744987595 0.195765328042533
## hsa-mir-122b-3p 10449.9542595749   1.25212835907867 0.293791801308695
## hsa-mir-15a-5p   696.18084962528 -0.677170624079328 0.162771620286646
## ...                          ...                ...               ...
## hsa-mir-769-5p  29.9692330488429 0.0653055737807121 0.340804461425901
## hsa-mir-942-5p  54.8480155832624 -0.591235768488896 0.325881717208209
## hsa-mir-95-3p    29.533154889069  0.691400207993933 0.351805136918705
## hsa-mir-4742-3p 17.8527070960152 0.0262833960823129 0.472512510966432
## hsa-mir-532-3p  23.5287803958615 -0.248081655266415 0.484669483704653
##                              stat               pvalue                 padj
##                         <numeric>            <numeric>            <numeric>
## hsa-mir-155-5p    5.35477200521163 8.56643641079564e-08 1.31066477085173e-05
## hsa-let-7c-5p     5.02206815847662 5.11180354684818e-07 3.91052971333886e-05
## hsa-let-7e-5p      4.8808221279102 1.05644496571581e-06 5.38786932515063e-05
## hsa-mir-122b-3p   4.26195814008787 2.02643407595029e-05 0.000775111034050985
## hsa-mir-15a-5p   -4.16024994336734 3.17899503523146e-05 0.000972772480780828
## ...                            ...                  ...                  ...
## hsa-mir-769-5p   0.191621827682297    0.848038443336644                   NA
## hsa-mir-942-5p   -1.81426492272701   0.0696369491588198                   NA
## hsa-mir-95-3p     1.96529309961071   0.0493803237896099                   NA
## hsa-mir-4742-3p  0.055624762249269    0.955640737618766                   NA
## hsa-mir-532-3p  -0.511857386543425    0.608750823822992                   NA
```

```
mcols(res)$description
```

```
## [1] "mean of normalized counts for all samples"
## [2] "log2 fold change (MLE): condition LS vs CTRL"
## [3] "standard error: condition LS vs CTRL"
## [4] "Wald statistic: condition LS vs CTRL"
## [5] "Wald test p-value: condition LS vs CTRL"
## [6] "BH adjusted p-values"
```

```
# Create a data frame of the ordered results (top 39)
padj.subset <- head(resOrdered, 40) %>%
 as_tibble(rownames = "miR")
```

```r
# Create a gene table of the subset
gt(padj.subset)
```

miR	baseMean	log2FoldChange	lfcSE	stat	pvalue	padj
hsa-mir-155-5p	649.46160	0.9050136	0.1690107	5.354772	8.566436e-08	1.310665e-05
hsa-let-7c-5p	978.12300	0.7287284	0.1451052	5.022068	5.111804e-07	3.910530e-05
hsa-let-7e-5p	859.89170	0.9554957	0.1957653	4.880822	1.056445e-06	5.387869e-05
hsa-mir-122b-3p	10449.95426	1.2521284	0.2937918	4.261958	2.026434e-05	7.751110e-04
hsa-mir-15a-5p	696.18085	-0.6771706	0.1627716	-4.160250	3.178995e-05	9.727725e-04
hsa-mir-185-5p	1357.82055	-0.4832699	0.1187742	-4.068813	4.725324e-05	1.204958e-03
hsa-mir-320a-3p	1364.86431	-0.7085482	0.1769815	-4.003515	6.240819e-05	1.364065e-03
hsa-mir-186-5p	695.68713	-0.5484831	0.1392865	-3.937805	8.223032e-05	1.572655e-03
hsa-let-7a-5p	52530.18452	0.5353146	0.1430846	3.741246	1.831105e-04	3.099959e-03
hsa-mir-10b-5p	377.10052	0.5000263	0.1354508	3.691571	2.228728e-04	3.099959e-03
hsa-mir-3613-5p	199.09014	-0.8803064	0.2374079	-3.707991	2.089098e-04	3.099959e-03
hsa-mir-22-3p	956.05588	-0.5218179	0.1447279	-3.605511	3.115393e-04	3.972126e-03
hsa-mir-19b-3p	635.12060	-0.4898627	0.1389969	-3.524271	4.246490e-04	4.997792e-03
hsa-mir-125a-5p	902.27728	0.4898229	0.1436390	3.410098	6.493953e-04	6.716305e-03
hsa-mir-451a	25224.85210	-0.7138156	0.2095565	-3.406315	6.584612e-04	6.716305e-03
hsa-mir-125b-5p	325.85157	0.5996200	0.1843262	3.253036	1.141789e-03	9.318571e-03
hsa-mir-15b-5p	332.37195	-0.5246529	0.1602709	-3.273538	1.062102e-03	9.318571e-03
hsa-mir-32-5p	335.88833	-0.5638403	0.1735308	-3.249223	1.157208e-03	9.318571e-03
hsa-mir-339-5p	140.38437	-0.8064061	0.2473712	-3.259903	1.114505e-03	9.318571e-03
hsa-mir-107	192.06085	-0.4640029	0.1462907	-3.171787	1.515041e-03	1.159006e-02
hsa-mir-484	537.21589	-0.7477914	0.2383122	-3.137865	1.701833e-03	1.239907e-02
hsa-let-7f-5p	26931.86012	0.3283732	0.1089469	3.014067	2.577710e-03	1.517213e-02
hsa-mir-206	286.97858	0.9944946	0.3286395	3.026096	2.477339e-03	1.517213e-02
hsa-mir-25-3p	5358.59661	-0.3748531	0.1243706	-3.014001	2.578270e-03	1.517213e-02
hsa-mir-27a-3p	680.69982	-0.3729175	0.1228875	-3.034626	2.408344e-03	1.517213e-02
hsa-mir-486-3p	318.76856	-0.5649174	0.1851226	-3.051585	2.276364e-03	1.517213e-02
hsa-mir-141-3p	168.69724	0.8738274	0.2923827	2.988643	2.802194e-03	1.587910e-02
hsa-mir-3074-5p	113.72153	-0.5372064	0.1851106	-2.902083	3.706899e-03	2.025555e-02
hsa-mir-126-3p	28299.34905	0.3275920	0.1144739	2.861718	4.213520e-03	2.148895e-02
hsa-mir-200a-3p	71.16591	0.8836924	0.3085261	2.864239	4.180130e-03	2.148895e-02
hsa-mir-221-3p	1840.17364	-0.3118071	0.1151012	-2.708981	6.749024e-03	3.330970e-02
hsa-mir-424-5p	123.37321	-0.6620369	0.2460831	-2.690298	7.138833e-03	3.413255e-02
hsa-let-7i-5p	24802.17441	0.2749761	0.1060285	2.593415	9.502795e-03	4.038688e-02
hsa-mir-23a-3p	1168.67494	-0.4371959	0.1683620	-2.596761	9.410726e-03	4.038688e-02
hsa-mir-27b-3p	1722.57673	0.4197088	0.1609568	2.607586	9.118305e-03	4.038688e-02
hsa-mir-486-5p	28527.14670	-0.4465920	0.1720814	-2.595236	9.452594e-03	4.038688e-02
hsa-mir-19a-3p	230.21461	-0.4406392	0.1742192	-2.529224	1.143152e-02	4.602690e-02
hsa-mir-222-3p	129.15396	-0.6472814	0.2554340	-2.534046	1.127540e-02	4.602690e-02
hsa-mir-363-3p	582.15551	-0.5365985	0.2149897	-2.495927	1.256285e-02	4.928501e-02
hsa-mir-92a-3p	14945.82469	-0.3695690	0.1487840	-2.483930	1.299413e-02	4.970254e-02

```r
# Create a data frame of all results for plotting
res.df <- as_tibble(res, rownames = "miR")

# Create a volcano plot of results
ggplot(data=res.df,
  aes(y=-log10(res$padj), x=res$log2FoldChange, text = paste(rownames(Counts)))) +
  xlab("Log2FC") +
```

```
ylab("-log10(Padj)") +
  geom_point(size=2) +
  geom_hline(yintercept = -log10(0.05), linetype="longdash", colour="grey", size=1) +
  geom_vline(xintercept = 1, linetype="longdash", colour="#BE684D", size=1) +
  geom_vline(xintercept = -1, linetype="longdash", colour="#2C467A", size=1) +
  labs(title="Volcano plot",
       subtitle = "LS healthy vs CTRL",
       caption=paste0("produced on ", Sys.time())) +
  theme_bw()
```

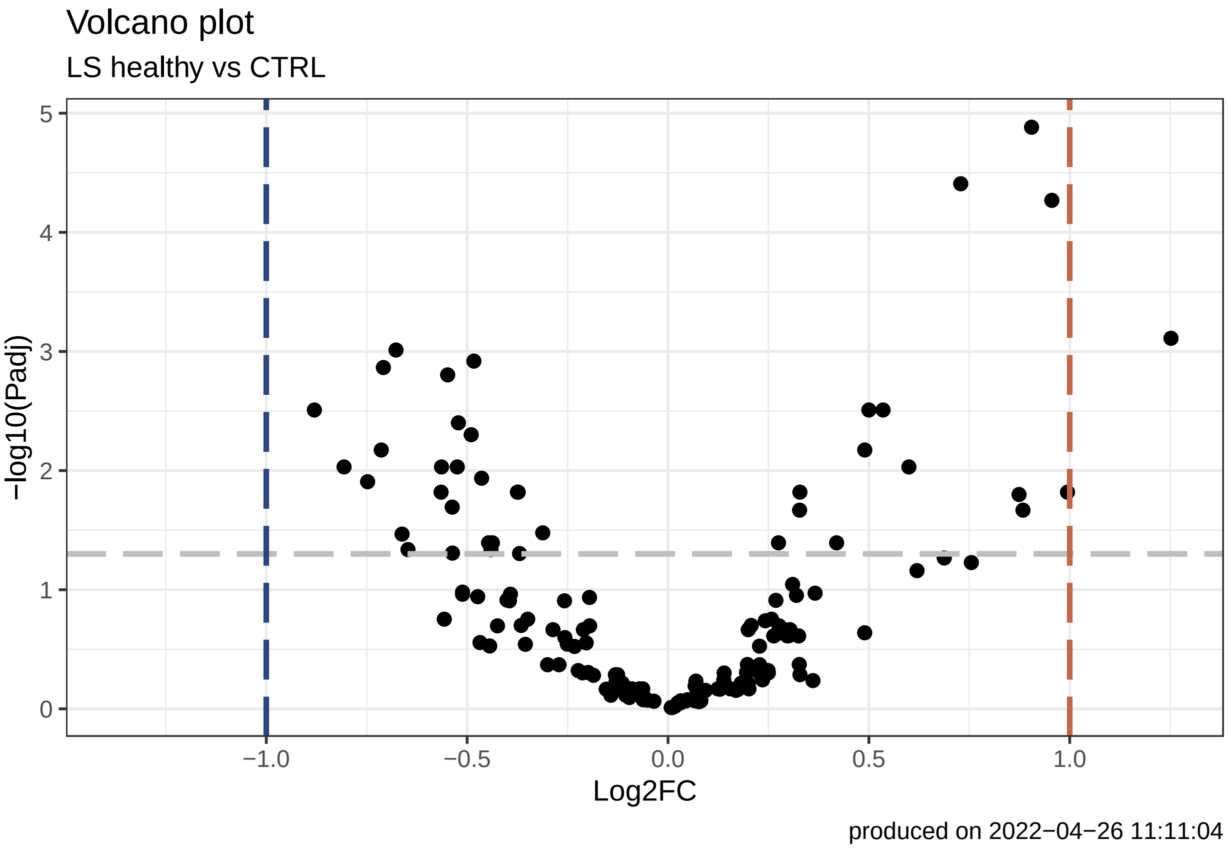

Step 7 - Sporadic rectal cancer patients vs healthy *path_MMR* carriers

This script is used to perform DE-analysis between SRME group and healthy *path_MMR* carriers.

```
# Choose conditions of interest (healthy path_MMR carriers, n = 81 and SRME, n = 24)
select <- which(targets$Type=="LS" & targets$Healthy_now=="YES" | targets$Type=="SRME")

# Create a new filtered counts file
Counts <- counts[,select]

# New phenofile with only the variables of interest
Targets <- targets[select,]

# Setup design matrix for DE-analysis
condition <- as.character (Targets$Type) # Condition of interest
```

```r
batch <- as.character (Targets$NGS) # Batch effect
sex <- as.character(Targets$Sex) # Sex as covariate
group_levels <- levels(as.factor(condition))
design <- data.frame(condition=as.factor(condition), batch=batch, sex=sex)
rownames(design) <- colnames(Counts)
dds <- DESeqDataSetFromMatrix(countData=Counts, colData=design, design = ~ batch + sex + condition)

# DESeq2 DE-analysis of the condition of interest, batch effect and sex as covariates
dds <- DESeq(dds)

# Display results
res <- results(dds, alpha=0.05)
resOrdered <- res[order(res$padj),]
summary(res)
```

```
##
## out of 228 with nonzero total read count
## adjusted p-value < 0.05
## LFC > 0 (up)       : 0, 0%
## LFC < 0 (down)     : 0, 0%
## outliers [1]       : 0, 0%
## low counts [2]     : 0, 0%
## (mean count < 9)
## [1] see 'cooksCutoff' argument of ?results
## [2] see 'independentFiltering' argument of ?results
```

```r
resOrdered
```

```
## log2 fold change (MLE): condition SRME vs LS
## Wald test p-value: condition SRME vs LS
## DataFrame with 228 rows and 6 columns
##                         baseMean       log2FoldChange             lfcSE
##                        <numeric>            <numeric>         <numeric>
## hsa-mir-10a-5p  415.729129524597    0.700061546934253 0.197350126933255
## hsa-mir-1180-3p 55.3191753024436      1.1469344634644 0.407027948054128
## hsa-mir-126-3p  27858.2897731659   -0.395198316713981 0.136574204015264
## hsa-mir-148b-3p 1429.34443333521   -0.335860304942317 0.111582302782957
## hsa-mir-196a-5p 32.2507910563298     1.41409583370707 0.497316069110564
## ...                          ...                  ...               ...
## hsa-mir-152-3p  101.315350736564  -0.0117083365991052 0.300777798273116
## hsa-mir-15a-5p  565.801351946751 -0.00508602975432469  0.19247823226543
## hsa-mir-19a-3p  200.176285402756  0.00281087665664761 0.231068789963995
## hsa-mir-20b-5p  118.924674916842  0.00375727754095451 0.266008834305326
## hsa-mir-485-3p  46.0863537963314   0.0134266672579918 0.608534438893608
##                               stat               pvalue               padj
##                          <numeric>            <numeric>          <numeric>
## hsa-mir-10a-5p     3.54730730510762 0.000389190337738628 0.0887353970044071
## hsa-mir-1180-3p     2.8178272989546  0.00483498082966978  0.157482232737816
## hsa-mir-126-3p    -2.89365271841388  0.00380789063706925  0.157482232737816
## hsa-mir-148b-3p   -3.00997825430804  0.00261266394498759  0.157482232737816
## hsa-mir-196a-5p    2.84345494050924   0.0044627319143887  0.157482232737816
## ...                             ...                  ...                ...
## hsa-mir-152-3p  -0.0389268645037214    0.968948698020339   0.99029423326846
## hsa-mir-15a-5p  -0.0264239217830668    0.978919213985837   0.99029423326846
## hsa-mir-19a-3p   0.0121646746714932     0.99029423326846   0.99029423326846
```

```
## hsa-mir-20b-5p   0.0141246344346665   0.988730546978105   0.99029423326846
## hsa-mir-485-3p   0.0220639398526124   0.982396951297894   0.99029423326846
```

```r
mcols(res)$description
```

```
## [1] "mean of normalized counts for all samples"
## [2] "log2 fold change (MLE): condition SRME vs LS"
## [3] "standard error: condition SRME vs LS"
## [4] "Wald statistic: condition SRME vs LS"
## [5] "Wald test p-value: condition SRME vs LS"
## [6] "BH adjusted p-values"
```

```r
# Create a data frame of the ordered results (top 20)
subset<- head(resOrdered, 20) %>%
  as_tibble(rownames = "miR")

# Create a gene table of the subset
gt(subset)
```

miR	baseMean	log2FoldChange	lfcSE	stat	pvalue	padj
hsa-mir-10a-5p	415.72913	0.7000615	0.1973501	3.547307	0.0003891903	0.0887354
hsa-mir-1180-3p	55.31918	1.1469345	0.4070279	2.817827	0.0048349808	0.1574822
hsa-mir-126-3p	27858.28977	-0.3951983	0.1365742	-2.893653	0.0038078906	0.1574822
hsa-mir-148b-3p	1429.34443	-0.3358603	0.1115823	-3.009978	0.0026126639	0.1574822
hsa-mir-196a-5p	32.25079	1.4140958	0.4973161	2.843455	0.0044627319	0.1574822
hsa-mir-320a-3p	1260.89967	0.5573208	0.1969468	2.829804	0.0046576534	0.1574822
hsa-mir-320b	138.33790	0.8445221	0.2851437	2.961742	0.0030590444	0.1574822
hsa-mir-486-5p	28352.51084	0.5415406	0.2062100	2.626161	0.0086354078	0.2461091
hsa-mir-320c	23.21182	1.0968594	0.4285908	2.559223	0.0104906547	0.2657633
hsa-mir-185-5p	1266.34806	0.3435518	0.1399213	2.455321	0.0140758737	0.2917545
hsa-mir-223-3p	36332.64595	0.4133965	0.1661891	2.487507	0.0128641845	0.2917545
hsa-mir-483-5p	260.32253	0.7739761	0.3242560	2.386929	0.0169897539	0.3228053
hsa-mir-2110	19.66771	1.1984973	0.5204457	2.302829	0.0212884818	0.3466981
hsa-mir-222-3p	117.27440	0.7502785	0.3226651	2.325255	0.0200583342	0.3466981
hsa-mir-486-3p	297.90130	0.4754586	0.2164429	2.196693	0.0280423622	0.4262439
hsa-let-7d-3p	227.49748	0.4615138	0.2285625	2.019201	0.0434662854	0.4677974
hsa-mir-11400	42.63983	-0.8238021	0.4118758	-2.000123	0.0454870007	0.4677974
hsa-mir-134-5p	120.39936	-0.6698154	0.3319645	-2.017732	0.0436192208	0.4677974
hsa-mir-193a-5p	94.50090	0.5318262	0.2682622	1.982487	0.0474247630	0.4677974
hsa-mir-196b-5p	316.86277	0.4468163	0.2272149	1.966492	0.0492418312	0.4677974

Step 8 - Sporadic rectal cancer patients vs *path_MMR* carriers with cancer

This script is used to perform DE-analysis between SRME group and *path_MMR* carriers with cancer.

```r
# Choose conditions of interest (path_MMR with cancer, n = 13 and SRME, n = 24)
select <- which(targets$Type=="LS" & targets$Healthy_now=="NO" | targets$Type=="SRME")

# Create a new filtered counts file
Counts <- counts[,select]

# New phenofile with only the variables of interest
Targets <- targets[select,]
```

```r
# Setup design matrix for DE-analysis
condition <- as.character (Targets$Type) # Condition of interest
batch <- as.character (Targets$NGS) # Batch effect
sex <- as.character(Targets$Sex) # Sex as covariate
group_levels <- levels(as.factor(condition))
design <- data.frame(condition=as.factor(condition), batch=batch, sex=sex)
rownames(design) <- colnames(Counts)
dds <- DESeqDataSetFromMatrix(countData=Counts, colData=design, design = ~ batch + sex + condition)

# DESeq2 DE-analysis of the condition of interest, batch effect and sex as covariates
dds <- DESeq(dds)

# Display results
res <- results(dds, alpha=0.05)
resOrdered <- res[order(res$padj),]
summary(res)
```

```
##
## out of 228 with nonzero total read count
## adjusted p-value < 0.05
## LFC > 0 (up)       : 0, 0%
## LFC < 0 (down)     : 0, 0%
## outliers [1]       : 0, 0%
## low counts [2]     : 0, 0%
## (mean count < 9)
## [1] see 'cooksCutoff' argument of ?results
## [2] see 'independentFiltering' argument of ?results
```

```r
resOrdered
```

```
## log2 fold change (MLE): condition SRME vs LS
## Wald test p-value: condition SRME vs LS
## DataFrame with 228 rows and 6 columns
##                         baseMean      log2FoldChange             lfcSE
##                        <numeric>           <numeric>         <numeric>
## hsa-let-7a-5p   60333.0739919861  0.0092484018664266 0.292951248812435
## hsa-let-7b-5p    101740.53065124  0.0649964919100044 0.278744679867905
## hsa-let-7c-5p   1191.71906684258   0.222884217723653 0.313235279614099
## hsa-let-7d-3p   277.106598697734   0.295007448481522 0.257524716257159
## hsa-let-7d-5p   1620.79876824715  -0.229396853580775 0.258804980000646
## ...                          ...                 ...               ...
## hsa-mir-99a-5p  111.185435654031   0.393622024916593 0.546722550230911
## hsa-mir-99b-5p    136.2175515144  0.0391449465983428 0.364010303946211
## hsa-mir-4742-3p 24.7866960150387    1.67461261850616  0.91933989739968
## hsa-mir-532-3p  35.3127536910979   -0.20117446613601 0.758781908425807
## hsa-mir-30e-5p  3943.14710049544 0.00187342365499215 0.227162529976706
##                               stat             pvalue              padj
##                          <numeric>          <numeric>         <numeric>
## hsa-let-7a-5p   0.0315697642659581  0.974815155986009 0.984058113657596
## hsa-let-7b-5p    0.233175721742226  0.815624957520626 0.984058113657596
## hsa-let-7c-5p    0.711555282017541  0.476740206505712 0.984058113657596
## hsa-let-7d-3p     1.14555003795027  0.251981380053937 0.984058113657596
## hsa-let-7d-5p    -0.88636954969028  0.375418422243555 0.984058113657596
## ...                            ...                ...               ...
```

```
## hsa-mir-99a-5p    0.719966690143557  0.471545504767019 0.984058113657596
## hsa-mir-99b-5p    0.107538017946127  0.914362166220587 0.984058113657596
## hsa-mir-4742-3p    1.82153806578257 0.0685251057836261 0.984058113657596
## hsa-mir-532-3p   -0.265128179654906   0.79091071965623 0.984058113657596
## hsa-mir-30e-5p  0.00824706281966599  0.993419870494506 0.993419870494506
```

```
mcols(res)$description
```

```
## [1] "mean of normalized counts for all samples"
## [2] "log2 fold change (MLE): condition SRME vs LS"
## [3] "standard error: condition SRME vs LS"
## [4] "Wald statistic: condition SRME vs LS"
## [5] "Wald test p-value: condition SRME vs LS"
## [6] "BH adjusted p-values"
```

```
# Create a data frame of the ordered results (top 20)
subset<- head(resOrdered, 20) %>%
  as_tibble(rownames = "miR")

# Create a gene table of the subset
gt(subset)
```

miR	baseMean	log2FoldChange	lfcSE	stat	pvalue	padj
hsa-let-7a-5p	60333.07399	0.009248402	0.2929512	0.03156976	0.97481516	0.9840581
hsa-let-7b-5p	101740.53065	0.064996492	0.2787447	0.23317572	0.81562496	0.9840581
hsa-let-7c-5p	1191.71907	0.222884218	0.3132353	0.71155528	0.47674021	0.9840581
hsa-let-7d-3p	277.10660	0.295007448	0.2575247	1.14555004	0.25198138	0.9840581
hsa-let-7d-5p	1620.79877	-0.229396854	0.2588050	-0.88636955	0.37541842	0.9840581
hsa-let-7e-5p	904.85522	0.260591230	0.3587440	0.72639881	0.46759432	0.9840581
hsa-let-7f-5p	28049.73885	0.236805612	0.2387517	0.99184886	0.32127126	0.9840581
hsa-let-7g-5p	4425.48657	0.194992567	0.2452425	0.79510113	0.42655468	0.9840581
hsa-let-7i-5p	25755.91770	-0.109233089	0.2233584	-0.48904839	0.62480744	0.9840581
hsa-mir-100-5p	56.87738	-0.585330355	0.5831189	-1.00379246	0.31547866	0.9840581
hsa-mir-101-3p	1060.15111	-0.255158402	0.3007912	-0.84829073	0.39627608	0.9840581
hsa-mir-103a-3p	2860.81663	0.135676208	0.2676541	0.50690881	0.61221880	0.9840581
hsa-mir-103b	985.95365	0.107224271	0.2608018	0.41113317	0.68097489	0.9840581
hsa-mir-106b-3p	254.61759	-0.592788554	0.3516875	-1.68555492	0.09188156	0.9840581
hsa-mir-106b-5p	81.11799	0.459848128	0.5790011	0.79420938	0.42707355	0.9840581
hsa-mir-107	172.92029	0.262385177	0.3688201	0.71141780	0.47682537	0.9840581
hsa-mir-10a-5p	563.55545	0.283853049	0.4081588	0.69544761	0.48677483	0.9840581
hsa-mir-10b-5p	465.30640	0.142811036	0.2315570	0.61674234	0.53740469	0.9840581
hsa-mir-11400	29.39506	-0.085038750	0.5911966	-0.14384174	0.88562544	0.9840581
hsa-mir-1180-3p	88.54225	0.075759366	0.4876652	0.15535118	0.87654447	0.9840581

Step 9 - Sporadic rectal cancer patients vs non-LS control group

This script is used to perform DE-analysis between SRME group and CTRL group.

```
# Choose conditions of interest (SRME, n = 24, CTRL, n = 37)
select <- which(targets$Type=="SRME" | targets$Type=="CTRL")

# Create a new filtered counts file
Counts <- counts[,select]
```

```r
# New phenofile with only the variables of interest
Targets <- targets[select,]

# Setup design matrix for DE-analysis
condition <- as.character (Targets$Type) # Condition of interest
batch <- as.character (Targets$NGS) # Batch effect
sex <- as.character(Targets$Sex) # Sex as covariate
group_levels <- levels(as.factor(condition))
design <- data.frame(condition=as.factor(condition), batch=batch, sex=sex)
rownames(design) <- colnames(Counts)
dds <- DESeqDataSetFromMatrix(countData=Counts, colData=design, design = ~ batch + sex + condition)

# DESeq2 DE-analysis of the condition of interest, batch effect and sex as covariates
dds <- DESeq(dds)

# Display results
res <- results(dds, alpha=0.05)
resOrdered <- res[order(res$padj),]
summary(res)
```

```
##
## out of 228 with nonzero total read count
## adjusted p-value < 0.05
## LFC > 0 (up)       : 4, 1.8%
## LFC < 0 (down)     : 0, 0%
## outliers [1]       : 0, 0%
## low counts [2]     : 0, 0%
## (mean count < 9)
## [1] see 'cooksCutoff' argument of ?results
## [2] see 'independentFiltering' argument of ?results
```

```r
resOrdered
```

```
## log2 fold change (MLE): condition SRME vs CTRL
## Wald test p-value: condition SRME vs CTRL
## DataFrame with 228 rows and 6 columns
##                         baseMean       log2FoldChange             lfcSE
##                        <numeric>            <numeric>         <numeric>
## hsa-mir-200a-3p 111.715251574488     1.75586404983053  0.37754305822285
## hsa-mir-10a-5p   466.81555503228    0.980500751171361 0.231783050278878
## hsa-mir-196a-5p 37.3750252312532     1.81268787183865 0.510932292180228
## hsa-mir-200c-3p 124.838948446616     1.13256759077687 0.324663822253165
## hsa-let-7e-5p   750.356684894863    0.721450941450189 0.236915042105674
## ...                          ...                  ...               ...
## hsa-let-7d-5p   1568.65433721735 -0.00574460659022131 0.148270261107422
## hsa-mir-3065-5p 33.7577867286205  0.00948004175628501  0.33179298733574
## hsa-mir-382-5p   466.84694089799  0.00765254387100355 0.299528556713819
## hsa-mir-106b-5p 93.2843324209975 -0.00414373615461178 0.352729339070495
## hsa-mir-4732-3p  36.556860995693  0.00312193397737304 0.431178830243722
##                             stat               pvalue                 padj
##                        <numeric>            <numeric>            <numeric>
## hsa-mir-200a-3p    4.65076502292383 3.30705960597195e-06 0.000754009590161605
## hsa-mir-10a-5p     4.23025216896419 2.33429489844175e-05  0.00266109618422359
## hsa-mir-196a-5p    3.54780447347265 0.000388456479126744    0.027694180338899
```

```
## hsa-mir-200c-3p    3.48843176587048 0.000485862812963141    0.027694180338899
## hsa-let-7e-5p      3.04518841453888  0.00232534584517635   0.0920451642655798
## ...                             ...                  ...                  ...
## hsa-let-7d-5p   -0.0387441591274959    0.969094365929624    0.986399622464081
## hsa-mir-3065-5p  0.0285721582978853    0.977205817475231    0.988286541248025
## hsa-mir-382-5p    0.025548628668201    0.979617361061639    0.988286541248025
## hsa-mir-106b-5p -0.0117476367730886    0.990626957584192    0.994222997555712
## hsa-mir-4732-3p 0.00724046209691783    0.994222997555712    0.994222997555712
```

```
mcols(res)$description
```

```
## [1] "mean of normalized counts for all samples"
## [2] "log2 fold change (MLE): condition SRME vs CTRL"
## [3] "standard error: condition SRME vs CTRL"
## [4] "Wald statistic: condition SRME vs CTRL"
## [5] "Wald test p-value: condition SRME vs CTRL"
## [6] "BH adjusted p-values"
```

```
# Create a data frame of the ordered results (top 3)
padj.subset<- head(resOrdered, 4) %>%
  as_tibble(rownames = "miR")

# Create a gene table of the subset
gt(padj.subset)
```

miR	baseMean	log2FoldChange	lfcSE	stat	pvalue	padj
hsa-mir-200a-3p	111.71525	1.7558640	0.3775431	4.650765	3.307060e-06	0.0007540096
hsa-mir-10a-5p	466.81556	0.9805008	0.2317831	4.230252	2.334295e-05	0.0026610962
hsa-mir-196a-5p	37.37503	1.8126879	0.5109323	3.547804	3.884565e-04	0.0276941803
hsa-mir-200c-3p	124.83895	1.1325676	0.3246638	3.488432	4.858628e-04	0.0276941803

```
# Create a data frame of all results for plotting
res.df <- as_tibble(res, rownames = "miR")

# Create a volcano plot of results
ggplot(data=res.df,
             aes(y=-log10(res$padj), x=res$log2FoldChange)) +
  xlab("Log2FC") +
  ylab("-log10(Padj)") +
  geom_point(size=2) +
  geom_hline(yintercept = -log10(0.05), linetype="longdash", colour="grey", size=1) +
  geom_vline(xintercept = 1, linetype="longdash", colour="#BE684D", size=1) +
  geom_vline(xintercept = -1, linetype="longdash", colour="#2C467A", size=1) +
  labs(title="Volcano plot",
       subtitle = "LS vs SRME",
       caption=paste0("produced on ", Sys.time())) +
  theme_bw()
```

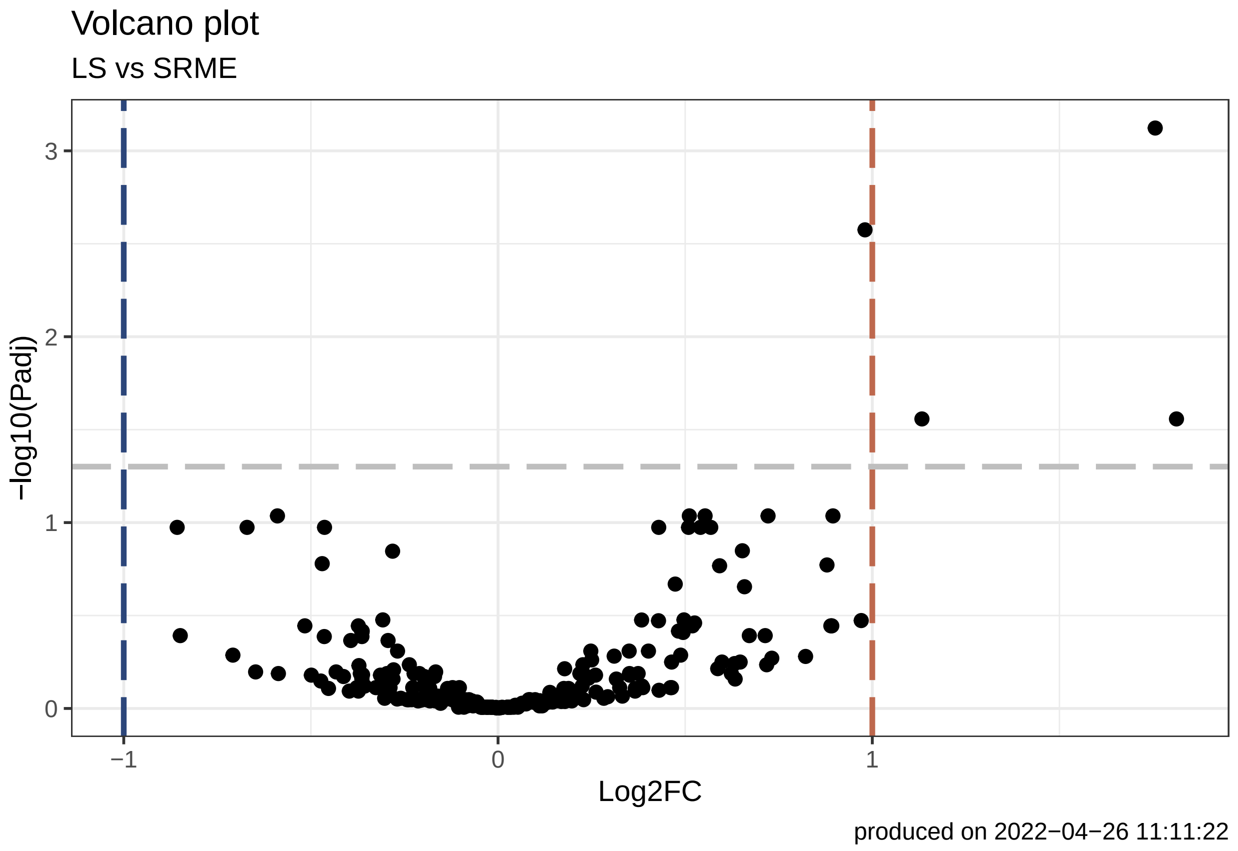

Step 10 - *Path_MMR* carriers with cancer vs non-LS control group

This script is used to perform DE-analysis between *path_MMR* carriers with cancer and CTRL group.

```
# Choose conditions of interest (Path_MMR carriers with cancer, n = 13 and CTRL, n = 37)
select <- which(targets$Type=="LS" & targets$Healthy_now=="NO" | targets$Type=="CTRL")

# Create a new filtered counts file
Counts <- counts[,select]

# New phenofile with only the variables of interest
Targets <- targets[select,]

# DE-analysis with DESeq2

# Setup design matrix for DE-analysis
condition <- as.character (Targets$Type) # condition of interest to be tested
batch <- as.character (Targets$NGS) # batch effect
sex <- as.character(Targets$Sex) # Sex as a covariate
group_levels <- levels(as.factor(condition))
design <- data.frame(condition=as.factor(condition), batch=batch, sex=sex)
rownames(design) <- colnames(Counts)
dds <- DESeqDataSetFromMatrix(countData=Counts, colData=design, design = ~ batch + sex + condition)

# DESeq2 DE-analysis of the condition of interest, batch effect taken into account, sex as a covariate
```

```r
dds <- DESeq(dds)

# Display results
res <- results(dds, alpha=0.05)
resOrdered <- res[order(res$padj),]
summary(res)
```

```
##
## out of 228 with nonzero total read count
## adjusted p-value < 0.05
## LFC > 0 (up)       : 0, 0%
## LFC < 0 (down)     : 0, 0%
## outliers [1]       : 0, 0%
## low counts [2]     : 0, 0%
## (mean count < 8)
## [1] see 'cooksCutoff' argument of ?results
## [2] see 'independentFiltering' argument of ?results
```

```r
resOrdered
```

```
## log2 fold change (MLE): condition LS vs CTRL
## Wald test p-value: condition LS vs CTRL
## DataFrame with 228 rows and 6 columns
##                         baseMean      log2FoldChange            lfcSE
##                        <numeric>           <numeric>        <numeric>
## hsa-mir-10b-5p  359.127945286884    0.687931059634368 0.237484888316919
## hsa-mir-125a-5p 780.501228445465    0.603420132306963 0.232331286457554
## hsa-mir-127-3p  45.1339945221773    -1.64300477204052 0.627080734315158
## hsa-mir-144-3p  1100.82460008364   -0.959114971832942 0.330034076855235
## hsa-mir-16-5p   371192.343384015    0.863315715176316 0.277753658945226
## ...                          ...                  ...               ...
## hsa-mir-96-5p    93.616441922745   0.0274689378713257 0.456928125815926
## hsa-mir-532-3p  25.1715112485986   0.0365344134704773 0.647935964977894
## hsa-mir-3529-3p 78.4982963166899   0.0126012560579685 0.352736564672473
## hsa-mir-28-3p   200.218788935536 -0.00291543605980014  0.34013614836977
## hsa-mir-425-5p  2271.73513458419  0.00221401883989526 0.185944771023344
##                                 stat              pvalue              padj
##                            <numeric>           <numeric>         <numeric>
## hsa-mir-10b-5p       2.8967361439703 0.00377066742205565 0.267832199418093
## hsa-mir-125a-5p     2.59724009412398 0.00939762103221378 0.267832199418093
## hsa-mir-127-3p     -2.62008491432106 0.00879078748750613 0.267832199418093
## hsa-mir-144-3p     -2.90610891145536 0.00365954048266793 0.267832199418093
## hsa-mir-16-5p       3.10820645335428 0.00188226529460209 0.267832199418093
## ...                              ...                 ...               ...
## hsa-mir-96-5p     0.0601165398218266    0.95206281691499 0.967768229771327
## hsa-mir-532-3p    0.0563858397206331   0.955034437274336 0.967768229771327
## hsa-mir-3529-3p   0.0357242693840633   0.971502218730709 0.980099583498238
## hsa-mir-28-3p   -0.00857137964833629   0.993161112254442 0.993161112254442
## hsa-mir-425-5p    0.0119068626007091   0.990499922640165 0.993161112254442
```

```r
mcols(res)$description
```

```
## [1] "mean of normalized counts for all samples"
## [2] "log2 fold change (MLE): condition LS vs CTRL"
## [3] "standard error: condition LS vs CTRL"
```

```
## [4] "Wald statistic: condition LS vs CTRL"
## [5] "Wald test p-value: condition LS vs CTRL"
## [6] "BH adjusted p-values"
```

```
# Create a data frame of the ordered results (top 20)
subset <- head(resOrdered, 20) %>%
  as_tibble(rownames = "miR")

# Create a gene table of the subset
#names(subset)<- c("miR","Mean", "log2FC", "SE", "Wald", "p-value", "FDR")
gt(subset)
```

miR	baseMean	log2FoldChange	lfcSE	stat	pvalue	padj
hsa-mir-10b-5p	359.12795	0.6879311	0.2374849	2.896736	0.003770667	0.2678322
hsa-mir-125a-5p	780.50123	0.6034201	0.2323313	2.597240	0.009397621	0.2678322
hsa-mir-127-3p	45.13399	-1.6430048	0.6270807	-2.620085	0.008790787	0.2678322
hsa-mir-144-3p	1100.82460	-0.9591150	0.3300341	-2.906109	0.003659540	0.2678322
hsa-mir-16-5p	371192.34338	0.8633157	0.2777537	3.108206	0.001882265	0.2678322
hsa-mir-32-5p	438.89304	-0.7494606	0.2812993	-2.664282	0.007715284	0.2678322
hsa-mir-361-3p	147.19465	0.9126396	0.3363962	2.712990	0.006667912	0.2678322
hsa-mir-423-3p	720.25553	-0.6033016	0.2136445	-2.823857	0.004744948	0.2678322
hsa-mir-15b-5p	402.36018	-0.7602167	0.3022087	-2.515535	0.011885187	0.2709823
hsa-mir-374a-5p	638.08518	-1.0141426	0.4004843	-2.532290	0.011332013	0.2709823
hsa-mir-181d-5p	40.64623	-1.2572744	0.5091163	-2.469523	0.013529335	0.2804262
hsa-mir-15a-5p	923.51193	-0.7358426	0.3083407	-2.386460	0.017011469	0.3216505
hsa-mir-190a-5p	364.32133	-0.6953374	0.3025734	-2.298078	0.021557323	0.3216505
hsa-mir-320a-3p	1690.77095	-0.6491387	0.2903951	-2.235364	0.025393458	0.3216505
hsa-mir-370-3p	48.76408	-1.2871668	0.5663018	-2.272934	0.023030145	0.3216505
hsa-mir-432-5p	561.06664	-0.9295053	0.4108023	-2.262659	0.023656737	0.3216505
hsa-mir-451a	37683.76940	-0.7295351	0.3252463	-2.243024	0.024895286	0.3216505
hsa-mir-654-3p	63.63660	-1.4009196	0.6009345	-2.331235	0.019740962	0.3216505
hsa-mir-107	229.77083	-0.6153741	0.2881726	-2.135436	0.032725443	0.3391546
hsa-mir-221-3p	2164.73228	-0.3891702	0.1820512	-2.137697	0.032541366	0.3391546

Session info

The output from running 'sessionInfo' is shown below and details all packages and version necessary to reproduce the results in this report.

```
sessionInfo()
```

```
## R version 3.6.3 (2020-02-29)
## Platform: x86_64-apple-darwin15.6.0 (64-bit)
## Running under: macOS  10.16
##
## Matrix products: default
## BLAS:   /Library/Frameworks/R.framework/Versions/3.6/Resources/lib/libRblas.0.dylib
## LAPACK: /Library/Frameworks/R.framework/Versions/3.6/Resources/lib/libRlapack.dylib
##
## locale:
## [1] fi_FI.UTF-8/fi_FI.UTF-8/fi_FI.UTF-8/C/fi_FI.UTF-8/fi_FI.UTF-8
##
## attached base packages:
## [1] parallel  stats4    stats     graphics  grDevices utils     datasets
```

```
## [8] methods   base
##
## other attached packages:
##  [1] DESeq2_1.26.0               SummarizedExperiment_1.16.1
##  [3] DelayedArray_0.12.3         BiocParallel_1.20.1
##  [5] matrixStats_0.61.0          Biobase_2.46.0
##  [7] GenomicRanges_1.38.0        GenomeInfoDb_1.22.1
##  [9] IRanges_2.20.2              S4Vectors_0.24.4
## [11] BiocGenerics_0.32.0         gt_0.4.0
## [13] forcats_0.5.1               stringr_1.4.0
## [15] dplyr_1.0.8                 purrr_0.3.4
## [17] readr_2.1.2                 tidyr_1.2.0
## [19] tibble_3.1.6                ggplot2_3.3.5
## [21] tidyverse_1.3.1             edgeR_3.28.1
## [23] limma_3.42.2                knitr_1.38
## [25] tinytex_0.38                rmarkdown_2.13
##
## loaded via a namespace (and not attached):
##  [1] colorspace_2.0-4       ellipsis_0.3.2         htmlTable_2.4.0
##  [4] XVector_0.26.0         base64enc_0.1-3        fs_1.5.2
##  [7] rstudioapi_0.13        farver_2.1.0           bit64_4.0.5
## [10] AnnotationDbi_1.48.0   fansi_1.0.3            lubridate_1.8.0
## [13] xml2_1.3.3             splines_3.6.3          cachem_1.0.6
## [16] geneplotter_1.64.0     Formula_1.2-4          jsonlite_1.8.0
## [19] broom_0.7.12           annotate_1.64.0        cluster_2.1.2
## [22] dbplyr_2.1.1           png_0.1-7              compiler_3.6.3
## [25] httr_1.4.2             backports_1.4.1        assertthat_0.2.1
## [28] Matrix_1.3-3           fastmap_1.1.0          cli_3.2.0
## [31] htmltools_0.5.2        tools_3.6.3            gtable_0.3.0
## [34] glue_1.6.2             GenomeInfoDbData_1.2.2 Rcpp_1.0.8.3
## [37] cellranger_1.1.0       vctrs_0.4.0            xfun_0.30
## [40] rvest_1.0.2            lifecycle_1.0.1        XML_3.99-0.3
## [43] zlibbioc_1.32.0        scales_1.1.1           hms_1.1.1
## [46] RColorBrewer_1.1-3     yaml_2.3.5             memoise_2.0.1
## [49] gridExtra_2.3          rpart_4.1.16           latticeExtra_0.6-29
## [52] stringi_1.7.6          RSQLite_2.2.11         highr_0.9
## [55] genefilter_1.68.0      checkmate_2.0.0        rlang_1.0.2
## [58] pkgconfig_2.0.3        bitops_1.0-7           evaluate_0.15
## [61] lattice_0.20-45        labeling_0.4.2         htmlwidgets_1.5.4
## [64] bit_4.0.4              tidyselect_1.1.2       magrittr_2.0.3
## [67] R6_2.5.1               generics_0.1.2         Hmisc_4.6-0
## [70] DBI_1.1.2              pillar_1.7.0           haven_2.4.3
## [73] foreign_0.8-75         withr_2.5.0            survival_3.3-1
## [76] RCurl_1.98-1.6         nnet_7.3-17            modelr_0.1.8
## [79] crayon_1.5.1           utf8_1.2.2             tzdb_0.3.0
## [82] jpeg_0.1-9             locfit_1.5-9.4         grid_3.6.3
## [85] readxl_1.4.0           data.table_1.14.2      blob_1.2.2
## [88] reprex_2.0.1           digest_0.6.29          xtable_1.8-6
## [91] munsell_0.5.0
```
